# Electric-Field-Driven Generative Nanoimprinting for Tilted Metasurface Nanostructures

**DOI:** 10.1007/s40820-025-01857-3

**Published:** 2025-07-28

**Authors:** Yu Fan, Chunhui Wang, Hongmiao Tian, Xiaoming Chen, Ben Q. Li, Zhaomin Wang, Xiangming Li, Xiaoliang Chen, Jinyou Shao

**Affiliations:** 1 Micro- and Nano-Technology Research Center, State Key Laboratory for Manufacturing Systems Engineering, Xi’an Jiaotong University, Xi’an, 710049 People’s Republic of China; 2 Frontier Institute of Science and Technology, and Interdisciplinary Research Center of Frontier Science and Technology, Xi’an Jiaotong University, Xi’an, 710049 People’s Republic of China; 3 Department of Mechanical Engineering, College of Engineering and Computer Science, University of Michigan-Dearborn, Dearborn, MI 48128 USA; 4 Mojie Technology Co., Ltd, Zhuhai, 519000 People’s Republic of China

**Keywords:** Generative nanoimprinting, Electric field assistance, Tilted metasurface structures, Large-area fabrication

## Abstract

Tilted metasurface nanostructures, with excellent physical properties and enormous application potential, pose an urgent need for manufacturing methods. Here, electric-field-driven generative-nanoimprinting technique is proposed. The electric field applied between the template and the substrate drives the contact, tilting, filling, and holding processes. By accurately controlling the introduced included angle between the flexible template and the substrate, tilted nanostructures with a controllable angle are imprinted onto the substrate, although they are vertical on the template. By flexibly adjusting the electric field intensity and the included angle, large-area uniform-tilted, gradient-tilted, and high-angle-tilted nanostructures are fabricated. In contrast to traditional replication, the morphology of the nanoimprinting structure is extended to customized control. This work provides a cost-effective, efficient, and versatile technology for the fabrication of various large-area tilted metasurface structures. As an illustration, a tilted nanograting with a high coupling efficiency is fabricated and integrated into augmented reality displays, demonstrating superior imaging quality.

**Supplementary Information:**

The online version contains supplementary material available at 10.1007/s40820-025-01857-3.

## Highlights

- The developed electric-field-driven generative-nanoimprinting technology enables direct fabrication of large-area tilted metasurface nanostructures with cost-efficiency and high-throughput advantages.
- Real-time tuning of process parameters facilitates customized fabrication of various tilted metasurface nanostructures.
- Integration of these custom-designed high-angle-tilted nanostructures into augmented reality displays achieves superior image quality.

**Supplementary Information:**

The online version contains supplementary material available at 10.1007/s40820-025-01857-3.

## Graphical abstract

**Figure:**
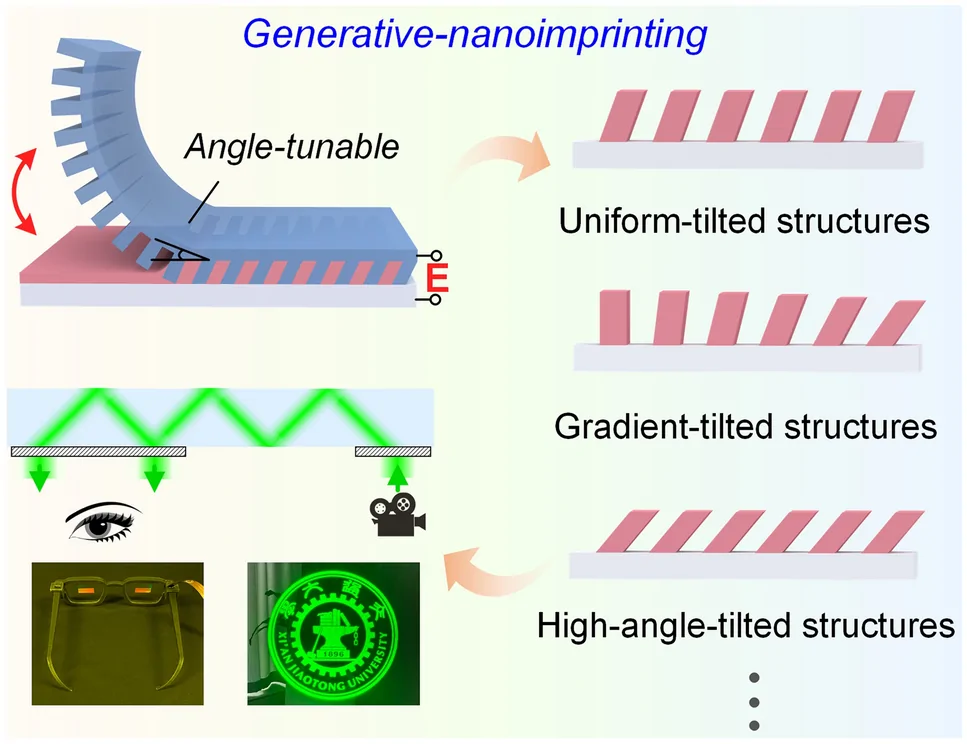

## Introduction

Tilted metasurface nanostructures, with their distinctive asymmetric geometric morphologies and characteristics, exhibit remarkable optical [1–3], electrical [4], magnetic [5, 6], and mechanical properties [7–9]. For example, based on tilted metasurface trapezoidal nanoholes arrays, Chen et al. observed intrinsic chiral bound states in the continuum for the first time [10]. These states markedly amplify the chiral interaction between light and matter, an interaction that has always been considered a fundamental topic in the cutting-edge field of chiroptics [11]. Beyond mechanistic study, the advantages exhibited by tilted structures in the development of high-performance devices in various fields, including photonics [12–14], sensing [15–17], displays [18, 19], biomedicine [20], and photovoltaics [21, 22], have been widely reported. Notably, subwavelength tilted gratings with a high coupling efficiency are widely recognized as the optimal optical solution for augmented reality (AR) displays [23, 24]—the next generation of intelligent terminals [25–29]. More novel theoretical mechanisms and higher-performance devices based on tilted metasurface nanostructures may be on the way.

The significant development potential of tilted metasurface nanostructures is propelling ongoing advancements and in-depth research into relevant fabrication methods. Currently, lithography techniques (such as extreme ultraviolet lithography and electron-beam lithography) and oblique etching (or oblique deposition) are the most commonly used methods for fabricating tilted structures [30–32]. Due to their intrinsic limitations, these approaches are only suitable for fabricating small-area tilted structures with fixed tilt angles. Moreover, such complex technological processes result in a low fabrication efficiency and high costs [33]. Secondly, methods for the direct fabrication of asymmetric nanostructures are also under investigation [34–36], such as laser machining [37] and ion beam cutting [38], which enables one-step fabrication, significantly simplifying the manufacturing process. Nevertheless, these serial fabrication methods are time-consuming and can only achieve a limited resolution. Thirdly, self-assembly technologies have also been employed for the fabrication of tilted nanostructures [39], but they result in the formation of numerous defects, rendering the obtained structures not suitable for application to high-precision fields, such as optical imaging. In addition, nanoimprinting also offers an effective method to replicate the tilted nanostructures from the template [40, 41]. Nevertheless, traditional nanoimprinting is merely a replication process, limited by the morphology of the mold [42–45]. The issues associated with this approach lie in the lithography process of the tilted master mold nanostructures. Although traditional nanoimprinting offers numerous advantages over the previously discussed approaches, such as low cost [46–49], high throughput [50–53], and high resolution [54–57], the nanoimprinting of large-area tilted nanostructures remains extremely challenging.

In this work, a novel technique, namely “electric-field-driven generative nanoimprinting” (E–G–N) is proposed, which enables the direct generation of controllable tilted metasurface nanostructures from an initial vertical template. An electric field is applied between the flexible template and the substrate. The entire forming process, including contact, tilting, filling, and holding, is driven by the electric field force. Different to the traditional nanoimprinting process, the contact process and state between the flexible template and the substrate are precisely controlled by introducing an included angle between the two. Therefore, the vertical nanostructures on the template come into contact with the substrate at a certain angle (rather than perpendicular contact) and are subsequently subjected to a controlled deflection to generate the desired tilted structure. The tilt angle of the metasurface nanostructures is controlled by adjusting the electric field intensity and the template release process. As verification, large-area uniform-tilted, gradient-tilted, and high-angle-tilted nanostructures have been sequentially generated and demonstrated. More customized morphology of tilted metasurface can be designed and developed based on the proposed process. Finally, a high-diffraction-efficiency tilted subwavelength grating was successfully developed and integrated into an AR displays, enabling the realization of a high-quality and high-brightness AR imaging display system.

## Experimental Section

### Nanoimprinting Process

The flexible working template with vertical nanostructures was obtained by replicating a silicon master mold. The preparation process was presented in detail in previous research [58]. Although this approach introduces a template deformation stage compared to traditional nanoimprinting techniques, the deformation remains small, controllable, and within the elastic deformation range, well below the material's fatigue limit threshold. The lifetime of the flexible templates in this method is comparable to that of traditional nanoimprinting techniques. Experimental validation has confirmed that they maintain excellent structural stability within 75 cycles (Fig. S1). Additionally, the working templates in the multistep nanoimprinting process were also fabricated using the abovementioned method.

The silicon master mold was prepared through electron-beam lithography, etching, and subsequent hydrophobic treatment. We selected silicon wafers and glass wafers as the nanoimprinting substrates. The silicon wafers were primarily used for structural SEM observation based on the ease with which they can be cut, while the glass wafers were utilized for sample display and diffraction efficiency testing. Before the experiments, the substrates were subjected to thermal treatment and adhesion promotion treatment (Section S2). It is noteworthy that the precise control of the resist thickness is essential in this process (Section S2). The generation of tilted metasurface structures fundamentally constitutes a force-induced deflection process, reliant on the contact counterforce from the substrate under electric field. This necessitates a dual requirement for thickness: sufficient to enable complete structure filling yet restrained to prevent residual layer thickening. Excessive thickness would cause buffering effects, impeding template–substrate contact and thus eliminating counterforce transmission, ultimately suppressing deformation initiation.

### Simulations

In this study, a comprehensive analysis of the deformation behavior of the flexible template was conducted using the ABAQUS software. As the template's structure layer thickness is smaller than that of the substrate, it was deemed appropriate to exclude the former from the simulation, and we concentrated instead on the polyethylene glycol terephthalate (PET) material of the substrate. The PET material had a Young's modulus of 2 GPa and a Poisson's ratio of 0.3. For computational simplification, the electric field force between the substrate and the template was converted into pressure. The elastic deformation of the nanostructures on the template was also analyzed using the ABAQUS software. Considering both the resolution of structure forming and the deformability during the generation of tilted structures, the Young's modulus of the working template was selected as 50 MPa. The optimization of the high-diffraction-efficiency tilted gratings for the wavelength of 532 nm and the study of the influence of the bending deformation on the diffraction efficiency were conducted using the R-SOFT software, where the refractive index of the material was set to 1.9.

### Characterization

All scanning electronic microscope (SEM) images were obtained using a Hitachi S-3000N scanning electron microscope. The optical performance of the AR displays was characterized using a comprehensive performance testing platform developed by Mojie Technology Co., Ltd.

## Results and Discussion

### Mechanism and Control Strategies of the E–G-N Technique for Tilted Nanostructures

**Fig. 1:**
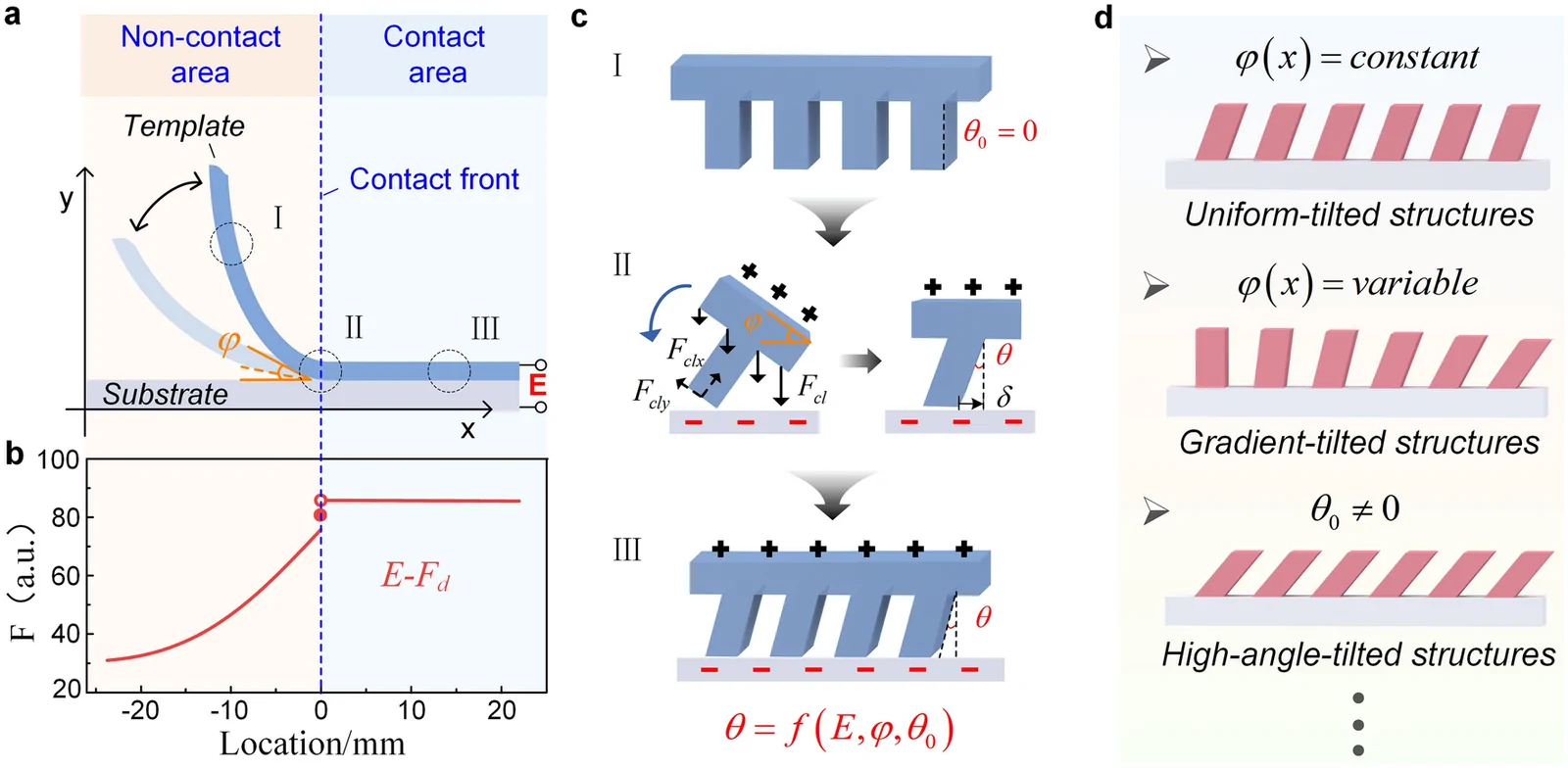
Schematics of the E–G-N method: **a** Schematic of the contact process and state between the template and substrate, **b** driving force of the template at different positions,** c** dynamic morphological evolution of the vertical structures on the template during the generation of tilted nanostructures, and **d** generation of various tilted metasurface nanostructures obtained by controlling the contact process and state

The core of the E–G–N method lies in the precise and flexible control of the contact process and state between the template and the substrate. The contact state is specifically characterized by the included angle (φ) between the template at the contact front and the substrate (Fig. 1a). This angle directly determines the contact state between the vertical structures on the template and the substrate and is thus a critical factor in generating tilted structures from the initial vertical structures. This method was carried out on a self-developed nanoimprinting device. The details and photographs of the equipment are provided in Fig. S2. The remarkable advantage of this equipment is that the relative position of the roller can be flexibly tuned, enabling the precise control of the included angle between the template and the substrate.

In this approach, the electric field (*E*) applied between the flexible template and the substrate induced surface/interfacial forces to drive the entire nanoimprinting process, including the contact, tilting, filling, and holding steps, which is different from pressure-based nanoimprinting. The driving force $E - F_{d}$ acting on the template mainly consists of the electrostatic attraction force $F_{ea}$ and the electric liquid bridge force $F_{eb}$. The distribution of these forces on the template is shown in Fig. 1b. In particular, $F_{ea}$ acts over the entire template and is inversely proportional to the distance between the two electrodes. $F_{eb}$ acts only on the wetted part of the template and mainly comprises the surface tension force $F_{eb}^{1}$ acting on the three-phase contact line and the liquid negative pressure force $F_{eb}^{2}$ acting on the wetted area [58]. The driving force acting on the contact line is $F_{cl}$:

1 $$F_{cl} = F_{ea} + F_{eb}^{1} = \frac{{\varepsilon_{r}^{2} \cdot \varepsilon_{0} }}{2}E^{2} A_{1} + \gamma l\sin \theta_{E} .$$

The driving force acting on the contact area is $F_{ca}$:

2 $$F_{ca} = F_{ea} + F_{eb}^{2} = \frac{{\varepsilon_{r}^{2} \cdot \varepsilon_{0} }}{2}E^{2} A_{1} + \frac{{2\gamma A_{2} \cos \theta_{E} }}{D}.$$

In the above equations, $\varepsilon_{r}$ represents the average dielectric constant of the dielectric layer between the two electrodes, $\varepsilon_{0}$ is the vacuum dielectric constant, *E* denotes the electric field intensity, $A_{1}$ is the area over which the electric field is applied, γ is the surface tension of the resist, $\theta_{E}$ represents the wetting angle of the resist after applying E, l is the length of the wetted part (that is the length of the three-phase line), $A_{2}$ represents the area of the two solid surfaces wetted by the liquid resist, and D is the distance between the two parallel plates. In the non-contact area, the maximum force is exerted at the contact line. This ensures that the template spreads in a line-contact manner, thereby preventing air bubbles from being trapped and achieving a complete nanoimprinting process.

The fabrication of the tilted nanostructures with the proposed method involves two steps: structure generation and state holding. Specifically, the generation of the tilted structures occurs at the contact line, where $F_{cl}$ provides the primary driving force. Behind the contact line, the combined action of $F_{ca}$ and the fluid resistance hold the template–substrate contact, thereby ensuring the stability of the tilted nanostructures. Figure 1c illustrates the morphological evolution of the vertical structures on the template during the fabrication process for different template regions (I–III). The direction in which the template spreads is perpendicular to the direction of the nanograting, with the structural filling and forming capability under these conditions particularly described (Section S4). Region I is the area where the template is not in contact with the substrate and the structures are vertical. Region II depicts the process of the vertical structures transforming into tilted structures at the contact line. Considering one nanograting period, then $A_{1} = 2d \cdot l$. Due to the included angle, the vertical structures on the template contact the substrate at an angle of φ. At the initial contact, the forces acting on the nanostructures are depicted in the figure. The force acts on the terminal end of the template nanograting structure. Under this driving force, the nanograting structure deflects, resulting in a lateral displacement of the terminal end of the grating, denoted as δ, and a corresponding tilt of the base of the grating by an angle θ, which results in the generation of the tilted structures. As the roller continues to move, the generated tilted structure follows rotate, with its top becoming horizontal. This process persists until the template is completely released, completing the nanoimprinting process for the entire sample. Region III represents the area behind the contact line, where the template–substrate contact is maintained due to the applied electric field and the fluid resistance of the resist, ensuring the stability of the tilted nanostructures. $F_{cl}$ is slightly smaller than $F_{ca}$. This minor difference between the two forces may lead to subtle differences in the generation and holding stage of the tilted structures. However, the difference between the two forces is so small that the resulting shape difference can be negligible. It is precisely the flexible controllability of the electric field that ensures the successful generation of the tilted structures; it would be challenging to fabricate such structures via the mechanical or fluid pressure method used in traditional nanoimprinting.

Based on the analysis of the generation process of tilted structures, it is evident that the generation of the tilted nanostructures results from the interplay between the included angle, electric field, and initial angle, namely $\theta { = }f\left( {E,\varphi ,\theta_{0} } \right)$. Therefore, its flexible fabrication ability breaking through the constraints of template morphology enables the generation of various tilted metasurface nanostructures through the precise control of the process parameters. As shown in Fig. 1d, when the included angle remains constant during the nanoimprinting process, large-area uniform-tilted nanostructures can be generated. In contrast, when the included angle dynamically changes, gradient-tilted nanostructures can be generated. Additionally, when a template with an initial tilt angle is used, that is, when the generated tilted structures serve as the basis for the next generation of tilted structures, high-angle-tilted nanostructures can be further generated. With further optimization of the contact state and process between the template and the substrate, the generation of a broader range of tilted nanostructures will become feasible.

**Fig. 2:**
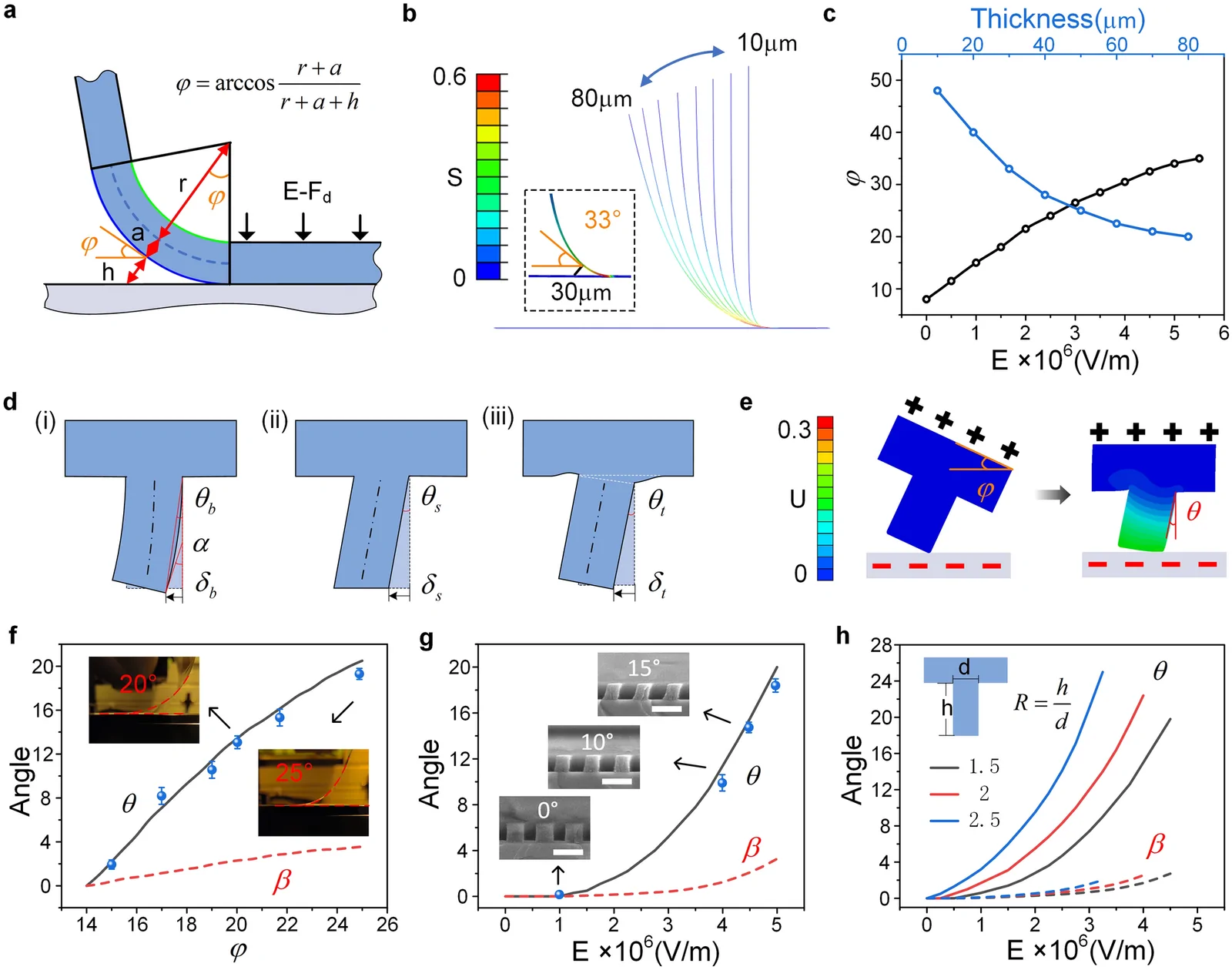
Analysis of the factors influencing the tilt angle of the nanostructures: **a** Simplified model of the included angle between the template and the substrate, **b** simulation model of the included angle of template with different thicknesses, **c** influence of the template thickness and electric field intensity on the included angle, **d** elastic deformation of the nanostructure, **e** simulation model of the generation process of the nanostructure, **f** influence of the included angle on the structure tilt and bending degrees (the insets display the photograph of different included angles), **g** influence of the electric field intensity on the structure tilt and bending degrees (the insets are SEM images of structures with different tilt angles under different electric field intensities, with a scale bar of 1 μm), and **h** influence of the aspect ratio (R) on the structure tilt and bending degrees

The control of the included angle between the template and the substrate is crucial for the generation of tilted structures. In this scheme, the angle can be adjusted by altering the position of the roller, but its adjustability range is jointly determined by the electric field intensity and the template thickness We conducted a detailed analysis of the adjustability range of the included angle. The bending behavior of the template can be simplified using the model shown in Fig. 2a. The right side of the template contacts the substrate under the electric field, and the left side of the template is constrained by the roller to be at a certain height from the substrate, thus forming an angle with the substrate. We define the angle between the tangent line to the top of the nanostructure and the substrate when the bottom of the nanostructure starts to contact the substrate as the included angle (φ). The expression in the inset shows that the included angle is related to the template thickness and the nanostructure height. In this expression, r represents the bending radius of the template, a denotes half of the template thickness, and h is the height of the structure. Compared to the template thickness, the height of the nanostructure can be considered negligible.

To study the adjustability range of the included angle, a simulation model was established. Figure 2b illustrates the maximum included angles that can be reached for different template thicknesses at *E* = 5 × 10^6^ V m^−1^. In particular, the inset shows that when the template thickness is 30 μm, the included angle is approximately 33°. This model is based on a prerequisite, that is, the contact between the right side of the template and the substrate, which is guaranteed by the electric field. The results are presented in Fig. 2c, where it can be seen that decreasing the template thickness and increasing the electric field intensity effectively broaden the range over which the included angle can be tuned.

In the nanoimprinting process, the deformation of the template nanostructure is crucial. Therefore, we conducted a detailed analysis of the stress-induced deformation of a single-period structure, whose details are provided in Section S5. As illustrated in Fig. 2d, the deformation of the structure mainly consists of three components, namely the bending deformation ($\delta_{b}$), shear deformation ($\delta_{s}$), and tilting of the substrate ($\delta_{t}$). Then, the total displacement of the nanostructure end (δ) is the sum of these three components. The tilt angle of the grating (θ) can be estimated as δ/h. In the bending deformation, the bending angle introduced by the shear load is unfavorable to the steepness of final formed structure. We carried out an analysis to assess the magnitude of the bending deformation during the generation of tilted structures. We denote the difference between the top turning angle (α) and the bottom tilt angle ($\theta_{b}$) of the grating as the bending angle β.

From the above force analysis, it is evident that the tilt and bending degrees of the structure are related to the included angle, electric field intensity, and aspect ratio of the structure. To further investigate the process and factors that influence the structure deformation, we established a simulation model to analyze its deformation process. The states of the template structure before and after deformation are shown in Fig. 2e, where the color bar represents the displacement (U). During the actual nanoimprinting process, the resist does play a certain buffering role in the deformation of the template structure. However, in this scheme, the resist layer thickness is relatively thin, the nanoimprinting speed is very slow, and the resist has a high degree of fluidity. As a result, this buffering effect is negligible. To simplify the simulation process, we neglected the impact of the resist on the template deformation and exclusively focused on the deformation of the template structure caused by the electric field force.

By combining finite element simulations with experimental measurements, we investigated the influence of the included angle (Fig. 2f), electric field intensity (Fig. 2g), and aspect ratio of the structures (Fig. 2h) on their tilt angle and bending angle. Comprehensive analytical details are provided in the Section S6. Further tilted structures with different tilt angles prepared under different electric field intensities are shown in Fig. S3. The results show that increasing these parameters leads to significantly larger tilt angles. Notably, the range of possible tilt angles of these nanostructures is limited. On the one hand, the three variables are constrained by certain limitations and cannot increase indefinitely. Firstly, an excessive electric field intensity can cause template breakdown and damage. Secondly, it is evident from the above analysis that the included angle can be adjusted within a certain range. Thirdly, the aspect ratio is constrained by the master mold. On the other hand, if the tilt and bending deformation exceed the material yield strength, the structure can be subjected to plastic deformation, which would affect the template lifespan. Indeed, as the tilt angle increases, the bending angle of the structures also increases, diminishing their steepness and potentially affecting their usability (Fig. S4). Therefore, an accurate process optimization should be performed by comprehensively evaluating and balancing the effects of the various processing parameters, structural characteristics, and performance requirements to meet the desired targets.

### Generation of Large-Area Uniform-Tilted Metasurface Nanostructures

**Fig. 3:**
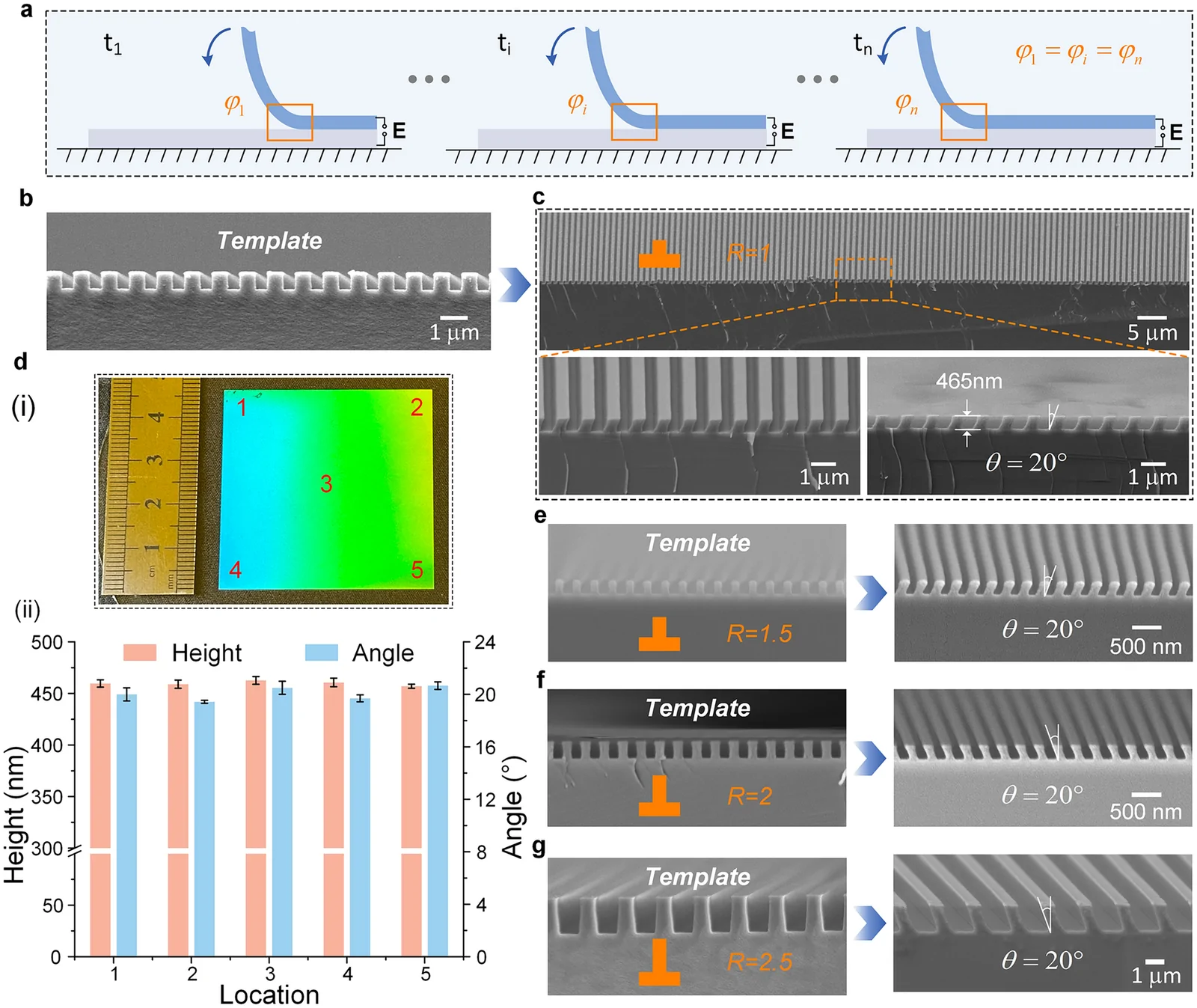
Generation of uniform-tilted metasurface nanostructures: **a** Schematic of the control process of the contact state during the generation of large-area uniform-tilted structures, **b** SEM image of a working template, **c** SEM images of uniform-tilted structures captured from different perspectives, **d** photograph showing the height and tilt angle of the structures at different locations (1–5) on the same sample, and **e–g** cross-sectional SEM images of templates with aspect ratios of 1.5, 2, and 2.5, alongside the corresponding generated uniform-tilted structures

During a single nanoimprinting process, the aspect ratio of the structures is fixed, and altering the electric field intensity is not advisable to prevent the occurrence of possible structural deformations. Therefore, adjusting the included angle is an effective way to precisely control the tilt angle of the structures. By keeping the included angle constant during the nanoimprinting process, the tilt angle of the structures generated at different times remains uniform (Fig. 3a). Following this approach, we generated uniform-tilted structures and conducted assessments of their integrity and uniformity.

Figure 3b illustrates the SEM image of a vertical structure working template with an aspect ratio of 1. Figure 3c presents the SEM images of large-area tilted nanostructures captured from different perspectives. As illustrated in the figure, the tilt angle of the structure is 20° (experimental parameters in Section S2). The structures exhibit exceptional straightness and sharpness across a wide area.

The uniformity of the structures is crucial when carrying out large-area nanoimprinting. We performed structural characterization at five different locations of the prepared sample, as illustrated in Fig. 3d-i. The corresponding SEM images are shown in Fig. S5. The dimensions of the large-area, tilted structure sample are 45 × 45 mm^2^. Three random test points were imaged at each location to determine the height and tilt angle of the tilted structure. The corresponding results are depicted in Fig. 3d-ii. It can be observed that both the height and tilt angle of the structures exhibit minimal fluctuations across a range of locations, which proves the excellent uniformity of the structures fabricated using this process even over large areas. This is attributed to the fact that the generation and holding stage of the tilted structure in this approach rely on the electric force, and there exists a causal relationship between the uniformity of the dielectric layer thickness of the template, the uniformity of the electric field, and the structural uniformity. The preparation process of the experimental template demonstrates that techniques like spraying, spin coating, and blade coating guarantee the uniformity of the dielectric layer thickness, thereby achieving uniformity in the electric field and ensuring the overall structural uniformity [58]. Furthermore, every stage of the template fabrication process and each step of the nanoimprinting process are compatible with semiconductor manufacturing techniques, and the uniformity and consistency metrics of each stage meet the requirements for large-scale production. Therefore, scaling this method to larger areas is fully feasible. Moreover, this method demonstrates excellent inter-sample dimensional uniformity during batch fabrication, further attesting to the stability of the technique (Fig. S6).

By precisely controlling the process parameters, vertical structures with different aspect ratios can be generated to customized tilted-angle nanostructures. We successfully fabricated 20° tilted nanostructures starting from vertical structures with aspect ratios of 1.5, 2, and 2.5 using the most suitable process parameters for each case. Figure 3e–g shows the cross-sectional SEM images of the vertical templates and the corresponding generated tilted structures (experimental parameters in Section S2). The images reveal that the tilted nanostructures exhibit excellent integrity and uniformity, confirming the suitability of this technique for use with different templates.

### Generation of Gradient-Tilted Metasurface Nanostructures

**Fig. 4:**
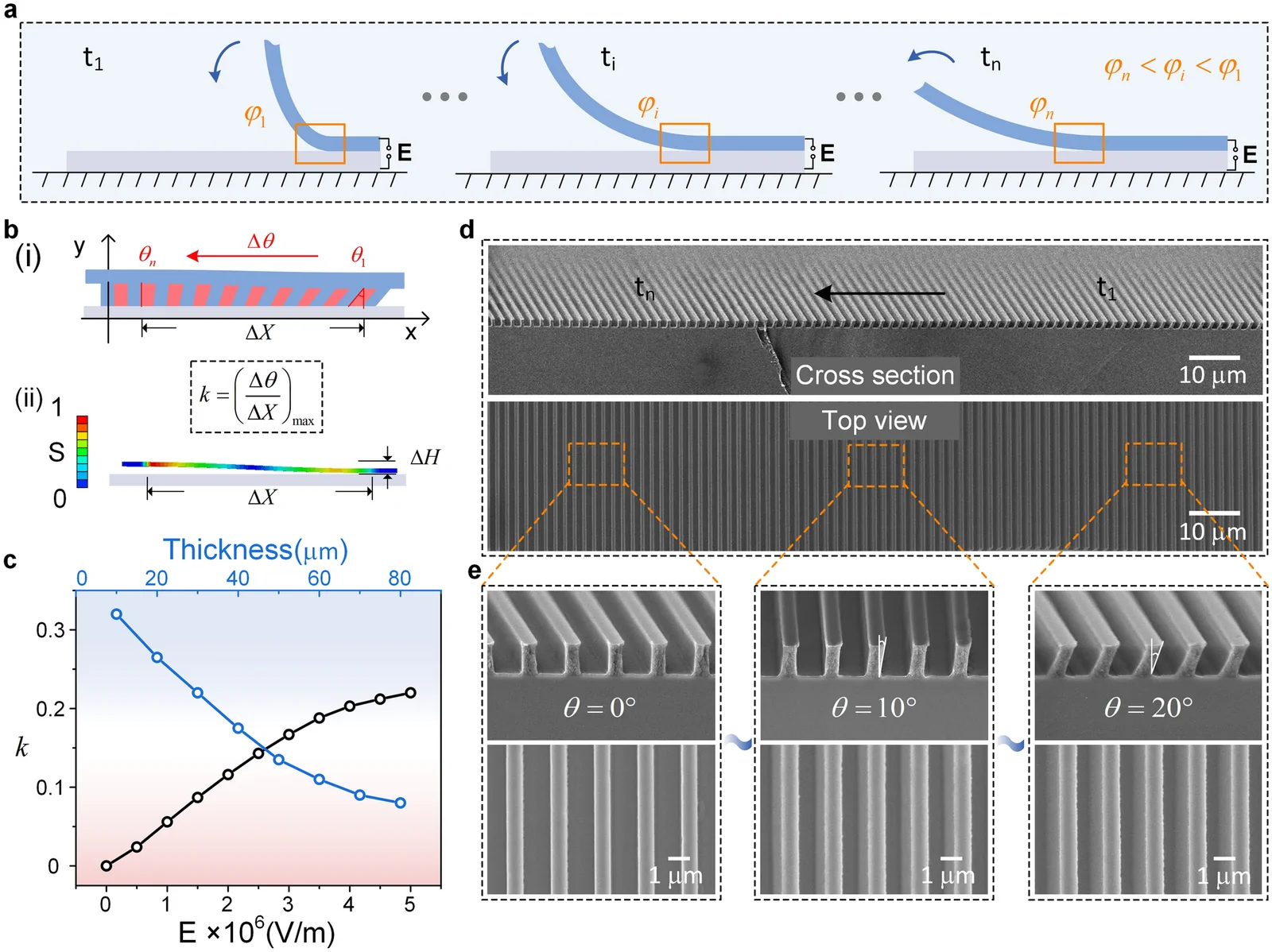
Generation of gradient-tilted metasurface nanostructures: **a** Schematic of the control process of the contact state during the generation of gradient-tilted structures, **b** rate of change of the tilt angle along the horizontal direction, **c** influence of the electric field intensity and template thickness on the maximum achievable rate of change of the tilt angle, **d** cross-sectional and top-view SEM images of obtained gradient-tilted structures, and **e** magnified SEM images of the tilted structures in different regions of the same sample showing different tilt angles

As previously discussed, the tilt angle can be effectively controlled by adjusting the included angle between the template and the substrate. Building on this principle, our approach enables also the generation of gradient-tilted nanostructures on a single substrate by tuning in real-time the included angle during a single-step nanoimprinting process. As shown in Fig. 4a, by gradually reducing the included angle, the tilt angle of the generated structures decreases gradually, and gradient-tilted nanostructures are thus obtained. Specifically, the included angle is adjusted by changing the position of the roller relative to the substrate. The generation process of the gradient-tilted structures is detailed in the Fig. S7.

The rate of change of the tilt angle of these gradient-tilted nanostructures in the horizontal direction is the focus of this study. We quantify this rate of change by determining the ratio of the tilt angle variation to the horizontal distance (expressed in degrees per micrometer) (Fig. 4b–i). A relatively small rate of change is easily achievable (e.g., 0 for uniform-tilted structures), so we focus on the maximum achievable rate of change (k). For a fixed electric field intensity and structural conditions, the included angle is the key factor influencing the tilt angle. Theoretically, as long as the included angle is within its tunability range, a corresponding tilt angle can be obtained. The required gradient-tilted angles can be achieved through reverse adjustment of the template-substrate included angle ($\Delta \theta = f(\Delta \varphi )$), enabling controlled fabrication of arbitrary gradient-tilted structures within the range of 0 to k. However, a change in the tilt angle leads to a bending deformation of the template in the horizontal direction, which is constrained by the material properties of the template; thus, the tilt angle cannot be changed to arbitrarily high values within a single period of the structure. Therefore, we study the rate of change of the tilt angle by examining the relationship between the deformation of the template and the horizontal distance (Fig. 4b ii). The bending deformation of the template is related to material properties such as the thickness of the template. Moreover, the template must maintain contact with the substrate after deformation, which is ensured by the electric field. Thus, the primary factors influencing the rate of change in tilt angle are the template thickness and the electric field intensity.

Figure 4c illustrates the influence of the electric field intensity and template thickness on the maximum achievable rate of change of the tilt angle. A higher electric field intensity enhances the force that drives the contact between the template and the substrate, enabling the template to deform over a smaller horizontal distance; consequently, k increases. Similarly, reducing the template thickness also results in an increase in k. Based on this, we can fabricate customizable gradient-tilted nanostructures.

Figure 4d shows the generated gradient-tilted nanostructures, which exhibit a smooth transition of the tilt angle from 20° to 0°, with the rate of change of the tilt angle is 0.16 (°/μm); these structures demonstrate excellent collimation and uniformity. We adopted the vertical structure as shown in Fig. 3g as the template. The included angle is 25° at t_1_, and is 10° at t_n_. *E* = 3 × 10^6^ V m^−1^. Figure 4e provides locally magnified views of three regions of the same sample with different tilt angles, each exhibiting perfectly formed structures with excellent sharpness.

Uniform-tilted and gradient-tilted nanostructures exhibit distinct light modulation characteristics. Through combined experimental and numerical simulations, we systematically investigated the reflectance and transmission responses of both structures across the visible light spectrum (Fig. S8). Notably, spatial variations in transmission/reflectance across gradient-tilted nanostructures confirm their capacity for cross-regional light amplitude modulation effects—a capability transcending traditional diffraction grating elements. Such gradient-tilted metasurface nanostructures enable light focusing, steering, large-area uniform emission, and achromatic operation through precise control of light amplitude or diffraction angles.

As a technique for generating/controlling tilted nanostructures, this method introduces additional manipulation strategies and parameters into metasurface design and fabrication. For unidirectional variable-sized nanostructures, its flexible control capabilities theoretically enable the realization of uniform-tilted or gradient-tilted metasurfaces. For rotation angle or other more complex metasurface structures, the tilt angle of unit structures can serve as an additional light modulation parameter alongside existing parameters (size, rotation angle), thereby expanding design degrees of freedom. During the design process of metasurface structures, the tilt angle of unit structures can be incorporated into the design parameter system. By treating the fabrication capability for structure tilt angles as a boundary condition and comprehensively considering the mutual coupling relationships among all parameters, multidimensional optical manipulation may be achieved, advancing the development of high-performance metasurface structures.

### Generation of High-Angle-Tilted Metasurface Nanostructures

**Fig. 5:**
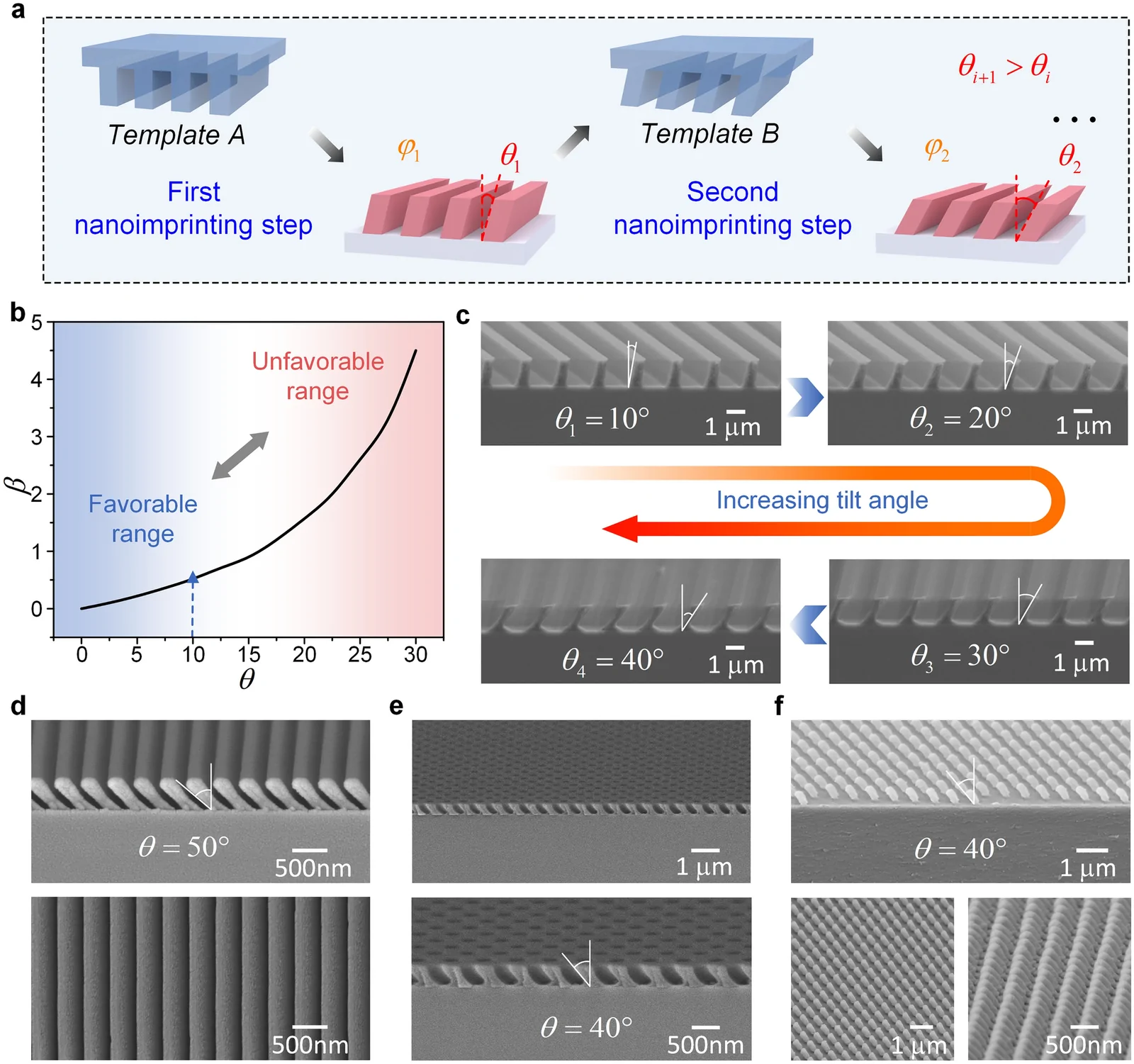
Generation of high-angle-tilted metasurface nanostructures: **a** Illustration of the multistep nanoimprinting process for generating high-angle-tilted structures, **b** relationship between the tilt angle and the bending angle during a single nanoimprinting step, **c** evolution of the tilt angle in structures fabricated via a four-step nanoimprinting process, alongside the corresponding cross-sectional SEM images, **d** tilted grating structure with a tilt angle of 50°, **e** tilted nanohole array structure with a tilt angle of 40°, and **f** replicated tilted nanopillar structure with an tilt angle of 40°

As revealed by the above discussion, the shear load introduced during the generation of the tilted structures inevitably induces a bending angle in the nanograting. Moreover, larger tilt angles correspond to greater bending angles, which is unacceptable for applications requiring high structural precision. To effectively mitigate the impact of the induced bending deformation and fabricate precise high-angle-tilted structures, we propose a multistep nanoimprinting process (Fig. 5a). By reducing the increment in the tilt angle in each nanoimprinting step and using the previously generated tilted structures as a template for the subsequent nanoimprinting step, we can dilute the influence of the bending deformation and generate high-angle-tilted nanostructures.

Figure 5b shows the relationship between the tilt angle and the bending angle of the tilted structures generated from vertical structures with an aspect ratio of 2.5. This figure shows that the bending angle increases nonlinearly with the tilt angle. Specifically, when the tilt angle is 10°, the bending angle is 0.5° (5% of the tilt angle); when the tilt angle is 20°, the bending angle is 8% of the tilt angle, indicating a superlinear increase. Therefore, it is more reasonable to select a smaller tilt angle for each nanoimprinting step during the multistep nanoimprinting process.

Figure 5c shows a tilted nanograting structure with a tilt angle of 40° fabricated through the proposed multistep nanoimprinting process with an increment in the tilt angle of 10° per nanoimprinting step. The bending angle can be effectively controlled to be ~ 5% (Section S2). Notably, while the multistep nanoimprinting processes can reduce the influence of the bending deformation on the obtained structures, utilizing too many sequential nanoimprinting steps is unsuitable, as the cumulative effect of several bending deformations may exceed the limit. Thus, multiobjective optimization is required in the design process of these high-angle-tilted nanostructures.

In this study, various high-angle-tilted metasurface nanostructures were generated using the multistep nanoimprinting process. The high-angle-tilted nanograting structures with a tilt angle of 50° were successfully generated (Fig. 5d). Figure 5e illustrates a nanohole array structure with a tilt angle of 40° (Fig. S9). Utilizing such nanohole structures as templates, we could also nanoimprint nanopillar array structures with a tilt angle of 40° (Fig. 5f). In summary, with the proposed technique, controllable generation of multidimensional, large-area, various high-angle-tilted metasurface nanostructures can be realized. Compared to other tilted nanostructures fabrication technique, the E–G–N technology demonstrates significant advantages in tilted-angle range, tilt diversity, cost, efficiency, and throughput while maintaining high resolution and structure uniformity, making it particularly suitable for industrial-scale deployment (Fig. S10).

### High-Performance AR Displays Based on Tilted Nanogratings

**Fig. 6:**
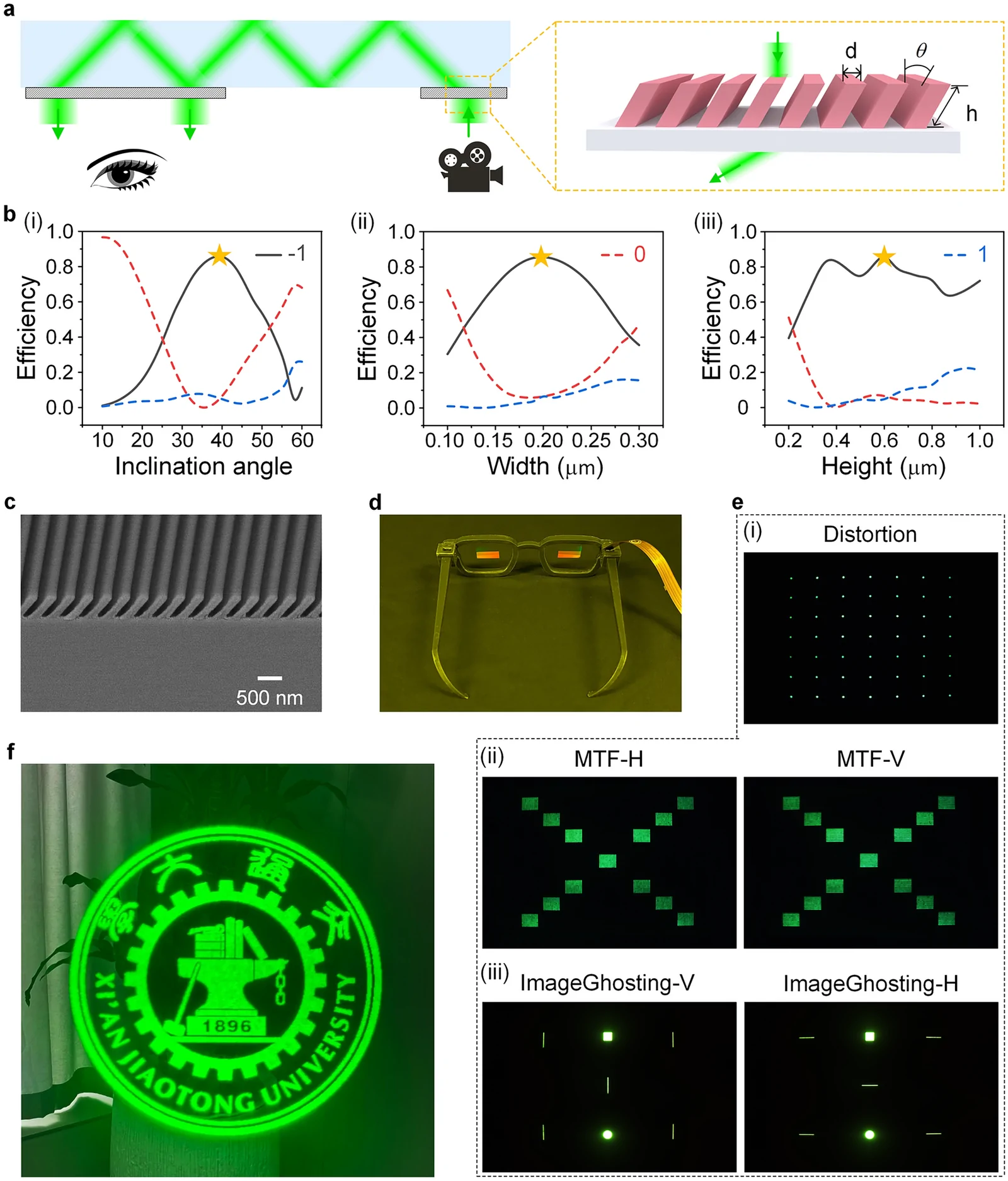
Tilted nanograting structures utilized in AR displays.** a** Schematic of a diffraction waveguide with surface relief gratings, **b** customized design of high-diffraction-efficiency tilted gratings, **c** SEM image of the tilted nanograting structure in the coupling-in region, **d** prototype of a pair of 3D printed AR glasses, **e** optical performance investigation of the prepared diffraction waveguide lenses, and **f** imaging performance of the developed AR displays

Tilted nanograting structures are widely used in diffractive waveguides due to their high diffraction efficiency, which renders them optimal structures for the coupling-in region in diffractive waveguides. The schematic of a diffractive waveguide is shown in Fig. 6a. By controlling parameters such as the tilt angle, width, and height of the tilted nanograting structures, the propagation angle and efficiency of the light can be precisely tailored [59–63].

To address the demand for high-diffraction-efficiency tilted gratings operating at a wavelength of 532 nm, we carefully optimized the structural parameters. The 532 nm wavelength has been selected as the design baseline wavelength for AR display, owing to its distinctive advantages in luminous efficiency and environmental adaptability. A grating with a period of 400 nm was selected, and simulations were conducted to study the influence of the tilt angle, width, and height of the grating on the diffraction efficiency. The simulation results are shown in Fig. 6b, which illustrates the variation in the diffraction efficiency of the − 1st, 0th, and + 1st order beams with the tilt angle, width, and height of the grating. It is evident that the structural parameters of the tilted grating that result in the highest diffraction efficiency are a tilt angle of 40°, a width of 200 nm, and a height of 600 nm. The design scheme of this functional structure not only provides a reference framework for designing analogous structures at other wavelengths, but also establishes feasibility foundation for developing and iterating full-color AR display technologies at the technical pathway level. Based on the above results, we fabricated an optimal tilted structure and successfully integrated it into a pair of AR glasses. The SEM image of the tilted grating structure is shown in Fig. 6c, which is consistent with the designed structure. In addition, to investigate the influence of the bending degree introduced during the generation of the grating on the final diffraction efficiency, we performed further simulations, the details of which can be found in Fig. S11. At a 2° bending angle of this tilted structure, the diffraction efficiency remains 98.5% of the original value, and thus, the impact of bending on the final result is not significant. The developed AR glasses are depicted in Fig. 6d. The AR waveguide lens is composed of an in-coupling region and an out-coupling region. Detailed geometric parameters and optical layout are presented in the Fig. S12. The glasses comprised diffractive waveguide lenses, a light engine, and a frame. The lenses were fabricated through the E–G–N process, while the frame was fabricated via 3D printing. The display of the light engine was controlled through an external driver, resulting in an overall compact structure.

A dedicated optical testing platform was employed to assess the performance of the waveguide lenses (Fig. 6e). Figure 6e-i shows the distortion of the image, with the four sides being distorted by 0.11%, 0.5%, 0.51%, and 0.52%. Figure 6e-ii shows the modulation transfer function (*MTF*) of the image, where the average *MTF* in the horizontal direction is 0.896, and that in the vertical direction is 0.87. Figure 6e-iii shows the ghosting of the image in the horizontal and vertical directions; the ghosting shift at the edge is 0, and that in the central region is 1.5. These comprehensive results indicate that the waveguide lenses with a tilted grating exhibit excellent display performance. Furthermore, through the application of image correction algorithms, the optical performance can be further enhanced. Figure 6f shows the image performance of the AR displays, from which it can be seen that the school emblem has clear lines and a high resolution. Compared with previously reported AR displays, the AR display developed in this work demonstrates significantly higher diffraction efficiency (Section S17). Furthermore, this approach enables scalable fabrication of AR displays while demonstrating exceptional reliability, establishing a solid foundation for future industrial applications (Fig. S13). These results not only confirm the application potential of the fabricated high-angle-tilted grating structures in AR displays but also provide valuable support for future optical design and performance optimization.

## Conclusions

In this study, we proposed an electric-field-driven generative-nanoimprinting technique, which enables the inexpensive and efficient fabrication of large-area, tilted metasurface nanostructures with tunable tilt angle. This approach permits the direct generation of technically challenging tilted nanostructures from simple vertical structures. We systematically analyzed the influence of the applied electric field intensity, included angle between the template and the substrate, and aspect ratio of the structures on the tilt angle of the obtained tilted nanostructures. By suitably controlling the relevant process parameters, various uniform-tilted, gradient-tilted, and high-angle-tilted nanostructures with different tilt angles were successfully generated; these unprecedented results cannot be achieved with the current fabrication processes. Moreover, the proposed method is suitable to fabricate more customized and various tilted metasurface nanostructures. The optimal parameter ranges determined through experiments and computational analysis provide guidance for fabricating standardized, large-scale, and multidimensional tilted structures. This work thus opens up new avenues for enhancing the performance of tilted structures and promoting their application in fields such as electronics, photonics, and mechanics.

## Supplementary Information

Below is the link to the electronic supplementary material.

Supplementary file1 (DOCX 7887 kb)
