# Supplementary material for: Electric-Field-Driven Generative Nanoimprinting for Tilted Metasurface Nanostructures
Source: Nanomicro Lett. 2025 Jul 28;18:12. doi: 10.1007/s40820-025-01857-3 (PMC12304336; doi:10.1007/s40820-025-01857-3)
Supplement: Supplementary file 1 — Supplementary file1 (DOCX 7887 kb) [file 40820_2025_1857_MOESM1_ESM.docx]

# Supporting Information for

**Electric-Field-Driven Generative-Nanoimprinting for Tilted Metasurface Nanostructures**

Yu Fan^1^, Chunhui Wang^1^, *, Hongmiao Tian^1^, Xiaoming Chen^1^, Ben Q. Li^1, 3^, Zhaomin Wang^4^, Xiangming Li^1, 2^, Xiaoliang Chen^1, 2^, and Jinyou Shao^1, 2^, *

^1^Micro- and Nano-technology Research Center, State Key Laboratory for Manufacturing Systems Engineering, Xi’an Jiaotong University, Xi’an 710049, P. R. China

^2^Frontier Institute of Science and Technology, and Interdisciplinary Research Center of Frontier science and technology, Xi’an Jiaotong University, Xi’an 710049, P. R. China

^3^Department of Mechanical Engineering, College of Engineering and Computer Science, University of Michigan-Dearborn, Dearborn, Michigan 48128, United States

^4^Mojie Technology Co., Ltd, Zhuhai 519000, P. R. China

*Corresponding authors. E-mail: [chw-nanoman@xjtu.edu.cn](mailto:chw-nanoman@xjtu.edu.cn) (Chunhui Wang); [jyshao@xjtu.edu.cn](mailto:jyshao@xjtu.edu.cn) (Jinyou Shao)

**S1 Lifetime of the flexible template**


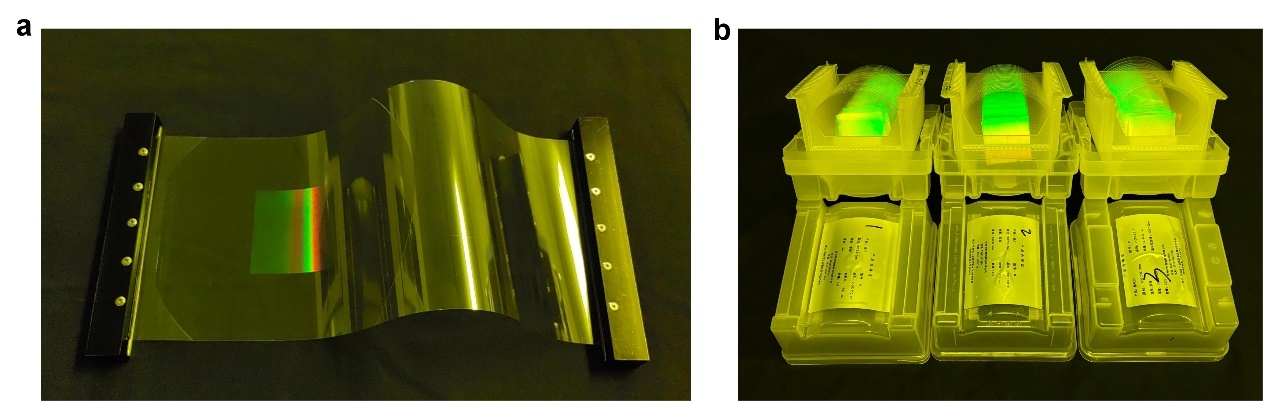


**Fig. S1** Lifetime of the flexible template**. a** Photograph of the flexible template, **b** samples fabricated via batch nanoimprinting using a flexible template.

**S2 Supplementary experimental section**

**S2.1 Preparatory processing of the substrate**

Before the experiments, the substrates were placed on a 200 ℃ hot plate and baked for 30 min to remove surface moisture and impurities, and they were then cooled to room temperature. Subsequently, an 8-nm-thick mr-APS1 resist was spin-coated onto the substrates at 5000 rpm for 60 s to serve as an adhesion promoter, and it was subsequently baked at 150 °C for 60 s to remove the solvent.

**S2.2 Thickness control of resist for different structures**

For the vertical structures with a linewidth of 500 nm, a period of 1 μm, and an aspect ratio of 1, we used a 500-nm-thick mr-UVcur21 resist that was spin-coated at 1000 rpm for 60 s and was then baked on an 80 °C hot plate for 60 s.

For the vertical structures with a line width of 150 nm, a period of 300 nm, and an aspect ratio of 1.5, a 120-nm-thick resist was spin-coated at 6000 rpm for 60 s.

For the vertical structures with a line width of 150 nm, a period of 300 nm, and an aspect ratio of 2, a 150-nm-thick resist was spin-coated at 5000 rpm for 60 s.

Finally, for the vertical structures with a line width of 700 nm, a period of 2 μm, and an aspect ratio of 2.5, a 1000-nm-thick resist was spin-coated at 500 rpm for 60 s. The mr-APS1 and mr-UVcur21 resists were purchased from Micro Resist Technology, GmbH.

**S2.3** **Experimental parameters for fabricating tilted structures with different aspect ratios**

We employed vertical structures with a line width of 500 nm, a period of 1 μm, and an aspect ratio of 1 to create tilted structures with a tilt angle of 20°, under the conditions of and $E=5\times10^{6}V/m$, with a bending angle of 3.3° (accounting for 16.35% of the tilt angle).

We employed vertical structures with a line width of 150 nm, a period of 300 nm, and an aspect ratio of 1.5 to create tilted structures with a tilt angle of 20°, under the conditions of and $E=4.5\times10^{6}V/m$, with a bending angle of 2.7° (accounting for 13.5% of the tilt angle).

In addition, we utilized vertical structures with a line width of 150 nm, a period of 300 nm, and an aspect ratio of 2 to fabricate tilted structures with a tilt angle of 20° under the conditions of and $E=4\times10^{6}V/m$, with a bending angle of 2° (accounting for 10% of the tilt angle).

We employed vertical structures with a line width of 700 nm, a period of 2 μm, and an aspect ratio of 2.5 to create tilted structures with a tilt angle of 20°, under the conditions of and $E=3\times10^{6}V/m$, with a bending angle of 1.6° (accounting for 8% of the tilt angle). The images reveal that the tilted nanostructures exhibit excellent integrity, and uniformity, confirming the suitability of this technique for use with different templates.

**S2.4** **Experimental parameters for fabricating high-angle tilted structures through the multistep nanoimprinting process**

The original working template A is composed of the vertical structure shown in **Fig. 3g**. Firstly, the tilted structures with a tilt angle of 10° were fabricated under an electric field intensity of $2\times10^{6}V/m$ and . Subsequently, working template B was replicated and utilized to fabricate tilted structures with a tilt angle of 20° under the conditions of and $E=1.5\times10^{6}V/m$. Since template B is tilted, it increases the component of the force that is perpendicular to the grating structure. Compared with template A, a smaller electric field can be applied to achieve the same tilt angle. Following the same steps, tilted nanograting structures with tilt angles of 30° and 40° were successively obtained. The bending angle can be effectively controlled to be ~5%.

**S3 Self-developed** **electric-field-driven generative-nanoimprint equipment**


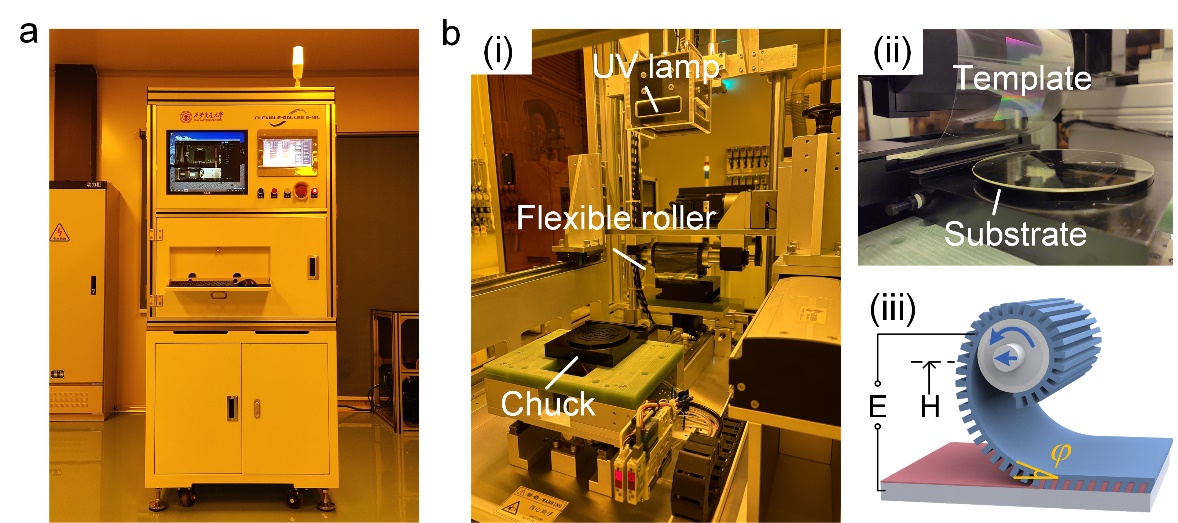


**Fig. S2** The electric-field-driven generative-nanoimprint equipment: **a** Photograph of the equipment's appearance, **b** photographs depicting the interior of the equipment as well as its key components

The E-G-N process is completed on the independently developed equipment. The general photograph of the equipment is depicted in **Fig. S2a**. This equipment consists of mechano-electronic system and control system, primarily targeting full-wafer nanoimprint on 4-inch wafers, with scalability to 6-inch and 8-inch wafer sizes. Among these, the flexible-roller is a crucial component in the nanoimprinting process, composed of a roller, a working template, and the chuck, as illustrated in **Fig. S2b**. The electric field is applied to the flexible template and the chuck that holds the substrate. One end of the flexible template is fixed on the substrate plane, while the other end is attached to a roller positioned at a certain height above the substrate. The remaining part of the template is smoothly wound around the roller, forming a flexible-roller. Through the rotation and movement of the roller, the template is gradually released and, driven by the electric field, progressively contacts the substrate to complete the nanoimprinting process. Significantly, the roller has the capability to move both horizontally and vertically. By doing so, it can alter its relative position with respect to the substrate, thereby adjusting the included angle between the template and the substrate. This enables precise control over the contact state between the template and the substrate.

**S4 Filling of the nanostructures**

It is important to note that in this approach, the spreading direction of the template is perpendicular to the direction of the nanograting. Compared with methods where the two directions are parallel, this approach presents several difficulties in expelling the trapped gas during structure filling. Based on previous research, when the template is oriented perpendicular to the direction of the nanograting, it has been found that the integrity of the structure depends on the viscosity of the liquid, the contact angle between the liquid and the template, and the spreading speed of the template^1, 2^. In our method, the viscosity of the resist is 2.2±0.2 mPa⋅s, the contact angle between the resist and the template is 17°, and the template is released at a speed of 1 mm/s; these values are comfortably within the optimal range of parameter values for achieving the complete structure formation. Moreover, larger included angles promote the release of gas, thereby preventing bubble entrapment and ensuring a high level of structural integrity.

**S5 Analysis of stress-induced deformation in nanostructures**

We take one period of the nanograting structure as the analysis object. Due to the included angle , the vertically downward driving force $F_{cl}$ received by the template at the contact line can be decomposed into perpendicular and parallel components, denoted as $F_{clx}$ and $F_{cly}$ respectively:

(S1)

(S2)

As shown in **Fig. 2d**, the torsional deformation of the nanostructure is primarily characterized by three components: bending deformation, shear deformation, and tilting of the substrate. Subsequently, we will conduct a detailed analysis of each deformation.

**S5.1** **Bending deformation**

When a force perpendicular to the direction of the nanostructure acts on the end of the structure, it induces bending deformation. We simplify the template as a cantilever beam. Under the sole influence of shear force, the displacement at the end of the grating can be expressed as follows:

$\delta_{b}^{0}=\frac{F_{clx}h^{3}}{3NI}$ (S3)

Where $F_{clx}$ represents the force acting perpendicular to the direction of the structure, $h$ denotes the height of the grating structure, N is the Young's modulus of the template material, and $I=\frac{ld^{3}}{12}$ is the second moment inertia.

In this scheme, the nanostructure experiences the combined effect of forces acting perpendicular to and parallel to the structure, with the parallel forces exacerbating this deformation. Thus, the total displacement of the grating end can be estimated as follows:

(S4)

Where represents the included angle, $d$ is the width of the structure, $F_{cr}=\frac{\pi^{2}\cdot N\cdot I}{\left(2h\right)^{2}}$ represents the critical buckling force when it is subjected only to normal force.

**S5.2** **Shear deformation**

When the template experiences shear force, it undergoes shear deformation, which can be expressed as follows:

$\delta_{s}=\frac{F_{clx}h}{KGA}$ (S5)

Where $K=\frac{6\left(1+\mu\right)}{7+6\mu}$ is the Timoshenko’s shear coefficient, $\mu$ is the Poisson ratio, $G=\frac{N}{2\left(1+\mu\right)}$ is the shear modulus, and $A=d\cdot l$ represents the cross-sectional area of the grating.

Therefore, the displacement of the shear deformation at the end of the grating is:

(S6)

**S5.3** **Tilting of the substrate**

When a force acts on the top of the nanostructure, it generates a moment $M$ at the bottom, resulting in an asymmetric stress distribution and subsequent tilted deformation.

$M=h\cdot F_{clx}$ (S7)

The maximum stress at the deformation site is:

$\delta_{\max}=8\frac{h}{d}\frac{F_{clx}}{A}$ (S8)

The rotation angle corresponding to the maximum stress is:

$\theta_{t}=\frac{T_{t}\delta_{\max}}{N}$ (S9)

Where $T_{t}=a\frac{1+\mu}{2\pi}\left\{2\left(1-\mu\right)+\left(1-\frac{1}{4\left(1-\mu\right)}\right)\right\}$ represents the tilting coefficient.

Therefore, the displacement at the end of the grating is:

(S10)

Combining the aforementioned analysis, the total displacement of the grating's end ($\delta$) can be represented as:

(S11)

The tilt angle of the grating, that is the rotation angle occurring at the bottom of the grating, can be represented as:

$\theta\cong\frac{\delta}{h}$ (S12)

In the bending deformation section, the grating lines undergo a certain bending degree. We simplify the template as a cantilever beam, and when it experiences only shear force, the rotation angle at the end of the grating lines is:

(S13)

In this section, the rotation angle at the bottom of the grating corresponding to the displacement at the end of the grating can be approximately expressed as:

$\theta_{b}\cong\frac{\delta_{b}}{h}$ (S14)

We use the difference between the two $\alpha-\theta_{b}$ to represent the bending degree of the grating:

(S15)

When $\beta=0$, it signifies purely inclined deformation without any bending degree. The larger the difference, the greater the bending degree of the grating.

**S6 Analysis of influencing factors on tilted angles of tilted nanostructures**

In this work, we selected a template with a thickness of 30 μm. The details and parameters of the nanoimprinting process are provided in the Materials and methods Section. As shown in **Fig. 2f**, for a vertical nanostructure (linewidth of 500 nm and aspect ratio of 1), when $E=5\times10^{6}V/m$, the deformation of the structure is not significant at a smaller included angle. However, at a higher included angle, upon increasing the included angle, the tilt and bending degrees of the structure also gradually increase. The data points in the figure correspond to the data derived from the nanoimprinting experiment, and the insets show photos of the templates with included angles of 20° and 25°. **Fig. 2g** demonstrates the influence of the electric field intensity on the tilt and bending degrees of the nanostructures (with an included angle of 25°, linewidth of 500 nm and aspect ratio of 1). As the electric field intensity increases, both the tilt and bending degrees increase. The insets depict the SEM images of tilted structures prepared at $E=1\times10^{6}V/m$, $E=4\times10^{6}V/m$, and $E=4.5\times10^{6}V/m$ with tilt angles of 0°, 10°, and 15°, respectively. **Fig. 2h** depicts the changes in the tilt and bending degrees of the structures with aspect ratios of 1.5, 2, and 2.5 at an included angle of 25° upon varying the electric field intensity. Larger aspect ratios lead to an increased tilt and bending deformation at a given electric field intensity and included angle.

**S7 Customized generation of the tilted structures using different electric field intensities**


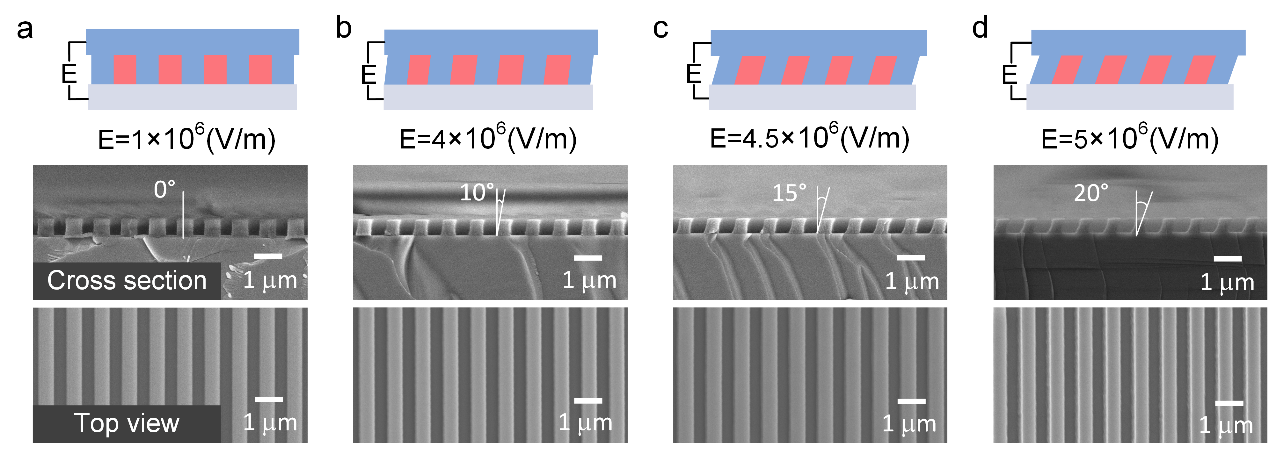


**Fig. S3** SEM images of tilted structures with different tilt angles under different electric field intensities

When the included angle is fixed, the tilted structures can be custom-generated by varying the electric field intensity. As shown in the figure above, for a working template with a vertical structure (with a line width of 500 nm, and an aspect ratio of 1) at the included angle of 25°, the tilted structures are presented under different electric field intensities. The greater the electric field intensity, the larger the tilt angle of the generated tilted structure, and correspondingly, the bending deformation also increases.

**S8 Excessive bending deformation of the structure**


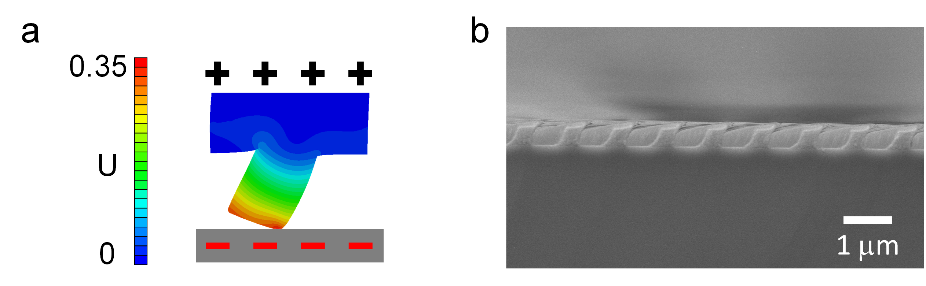


**Fig. S4** Simulation model of excessive bending deformation of the structure and SEM cross-sectional image

When the electric field intensity and the included angle are too large, although the tilt angle of the grating increases, the bending deformation of the structure also significantly increases, thereby affecting the fidelity of the structure. As shown in the **Fig. S4**, the simulation model of the structural deformation when $E=6\times10^{6}V/m$, is depicted on the left, with the SEM cross-sectional view of the structure shown on the right. It can be observed from the figure that not only does the bending deformation of the structure increase, but also deformations occur at the top and bottom of the structure, which is clearly not the desired outcome. Thus, it is important to regulate grating deformation during the fabrication of tilted structures.

**S9 Uniformity of the sample tilted structures**


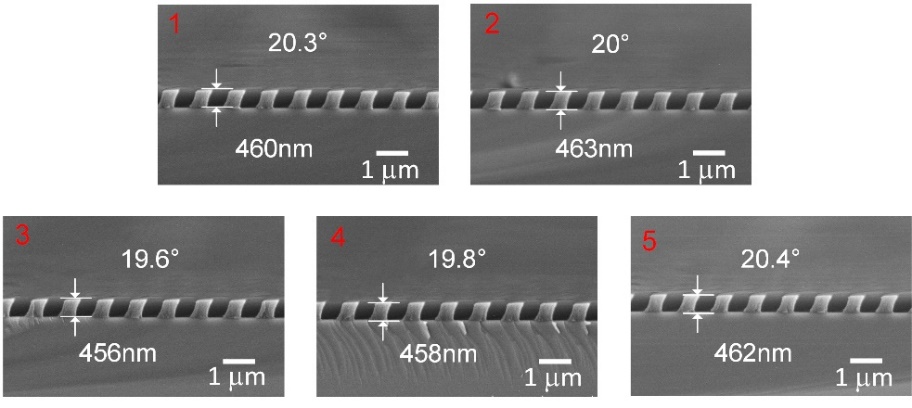


**Fig. S5** SEM cross-sectional images and dimensions of the tilted gratings at different positions

**Fig. S5** presents SEM images of the structures at five different positions within the sample area. Annotations denote the tilt angle and height of the tilted nanograting structures. The figure illustrates minimal variation in structure dimensions across different positions.

**S10 Inter-sample uniformity of structure dimensions**


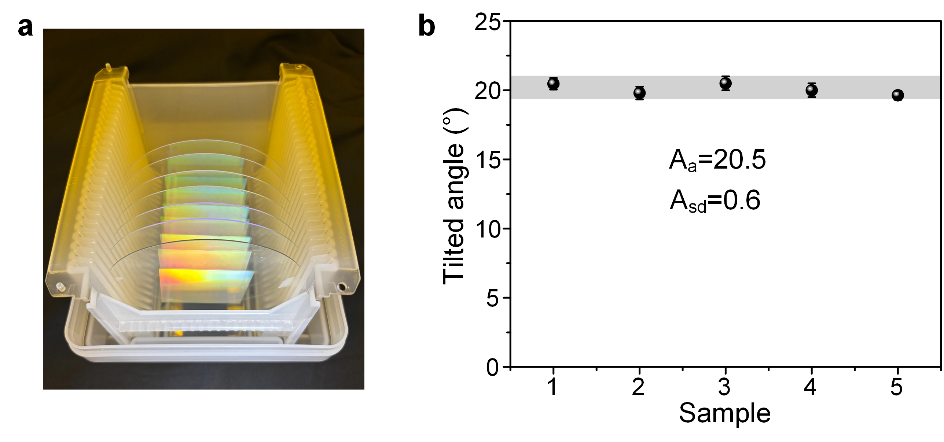


**Fig. S6** Inter-sample uniformity of structure dimensions. The results indicate excellent consistency in tilt angles across different samples, with an average value of 20.5° and a standard deviation of 0.6. Inter-sample consistency remains within 1.5%, validating the stability of the fabrication process

**S11 Generation process of the gradient-tilted structures**

**
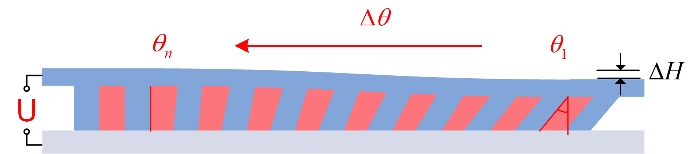
**

**Fig. S7** Schematic of the gradient-tilted nanostructures

During the generation process of gradient-tilted nanostructures, the tilt angle of the generated structures can be adjusted by varying the included angle between the template and the substrate. At the contact line, due to the differences in the included angles, the generated tilted structures exhibit different tilt angles. In practice, the electric field between the template and the substrate is established by applying a voltage across them. Behind the contact line, the different tilt angles lead to subtle differences in the vertical height of the structures, which in turn leads to variations in the electric field intensities. As a result, $F_{ca}$ differs in regions with different tilt angles. Specifically, the larger the tilt angle, the greater $F_{ca}$. This gradient-distributed force and the fluid resistance jointly provides mechanical support for the nanostructures in regions with different tilt angles, ensuring the relative stability of the tilted structures in each region until the nanoimprinting process is completed

**S12 Light modulation effects of different tilted metasurface nanostructures**

**
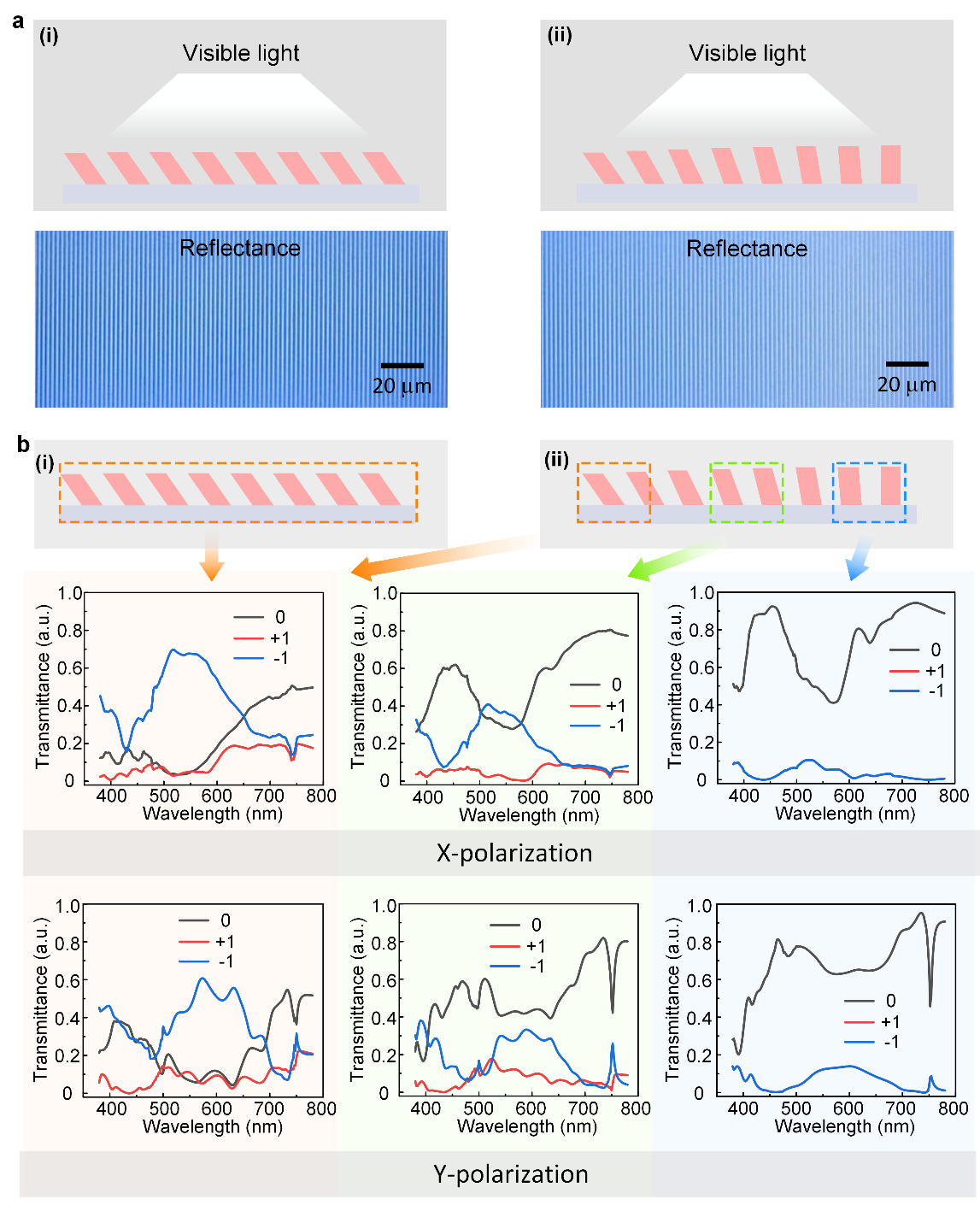
**

**Fig. S8 Light modulation effects of different tilted nanostructures.** **a** Reflectance characteristics of uniform-tilted and gradient-tilted metasurface nanostructures, **b** numerical simulation of transmission properties

**S13 Multistep nanoimprinting processes for generating high-angle-tilted nanohole arrays**

**
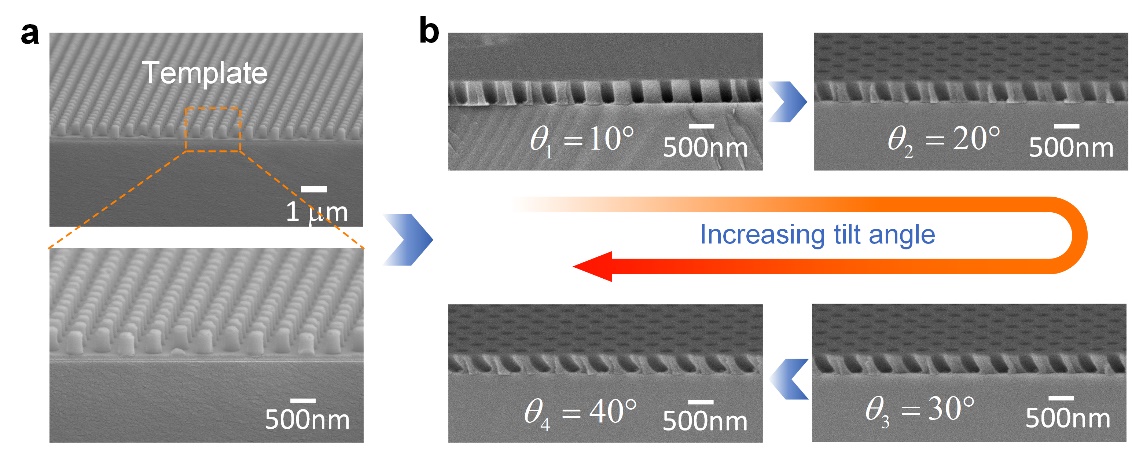
**

**Fig. S9** The vertical structure of the template and the evolution process of the tilted structures during multistep nanoimprinting for generating the high-angle nanohole arrays

The original working template A, which consists of the vertical nanopillar array structure (diameter of 300 nm, period of 600 nm, and height of 500 nm), is shown in **Fig. S9a**. **Figure S9b** illustrates the high-angle-tilted nanohole structures fabricated through multi-step nanoimprinting process. Firstly, the tilted structures with an angle of 10° were fabricated under the electric field intensity of $1.5\times10^{6}V/m$, with . Subsequently, the working template B was replicated by taking the tilted structure obtained in the first nanoimprinting step as the master mold. The working template B was then utilized to fabricate tilted structures with an angle of 20° under the conditions of and $E=1\times10^{6}V/m$. Following the same steps, tilted nanohole structures with tilt angles of 30° and 40° were obtained successively.

**S14 Comparison of different techniques for tilted nanostructures fabrication**

A quantitative comparison of key fabrication capabilities (including tilted angle, resolution, processing area, processing time, cost, and uniformity) is presented between the proposed E-G-N technology and existing techniques for tilted nanostructure fabrication (e.g., oblique etching, laser machining, ion beam cutting), as shown in **Fig. S10**. The corresponding data, derived from **Table S1**, have been normalized to clearly demonstrate the relative magnitudes of the parameters. The E-G-N technique employs a multi-step nanoimprinting process capable of fabricating nanostructures with high-tilted angles. Structures with tilt angles up to 50° were demonstrated in this work. By utilizing templates with higher aspect ratios or increasing the number of nanoimprinting steps, tilt angles could be extended to 60–70°. Additionally, the vertically structure templates (fabricated via electron-beam lithography) enable a resolution of <50 nm. While the demonstrated sample area in this study is 45 mm*45 mm, the processing platform (**Fig. S1**) supports scalable expansion for larger-area fabrication.


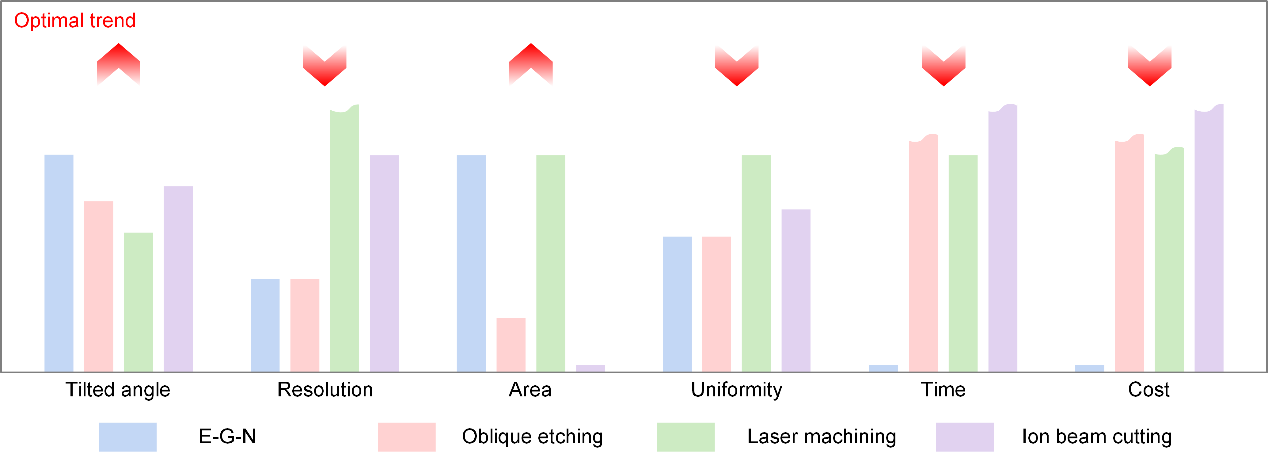


**Fig. S10** Comparison of different techniques for tilted nanostructures fabrication

In summary, the E-G-N technology demonstrates significant advantages in tilted-angle range, tilt diversity, cost, efficiency, and throughput while maintaining high resolution and structure uniformity, making it particularly suitable for industrial-scale deployment.

**Table S1** Fabrication capabilities: proposed E-G-N versus other tilted-nanostructures fabrication techniques.

| Techniques | Tilted angle | Resolution | Area | References |
| --- | --- | --- | --- | --- |
| E-G-N | 50°  (60°-70°) | 150 nm  (<50 nm) | 45 mm*45 mm  (>50 mm) | ~ |
| Oblique etching | 55° | 380 nm | 10 mm*10 mm | [S3] |
|  | 45° | 100 nm | ~ | [S4] |
|  | 45° | 30 nm | 4.7-8 | [S5] |
|  | 12° | 40 nm | 15 mm*15 mm | [S6] |
|  | 20° | 345 nm | 15 mm*15 mm | [S7] |
|  | 45° | 300 nm | 1 mm*1 mm | [S8] |
|  | 45° | 260 nm | 3 mm*3 mm | [S9] |
|  | 35° | 200 nm | 4 mm*4 mm | [S10] |
| Laser machining | 30° | 1 μm | ~ | [S11] |
|  | 30° | 24 μm | ~ | [S12] |
|  | 3° | 10 μm | ~ | [S13] |
|  | 45° | 4.6 μm | ~ | [S14] |
| Ion beam cutting | 60° | 108 nm | ~ | [S15] |
|  | 45° | 80 nm | 50 μm*50 μm | [S16] |
|  | 46° | 70 nm | 15 μm*15 μm | [S17] |
|  | 40° | 85 nm | 7 μm*7 μm | [S18] |

**S15 Influence of bending deformation on the diffraction efficiency of the tilted nanograting structures**

**
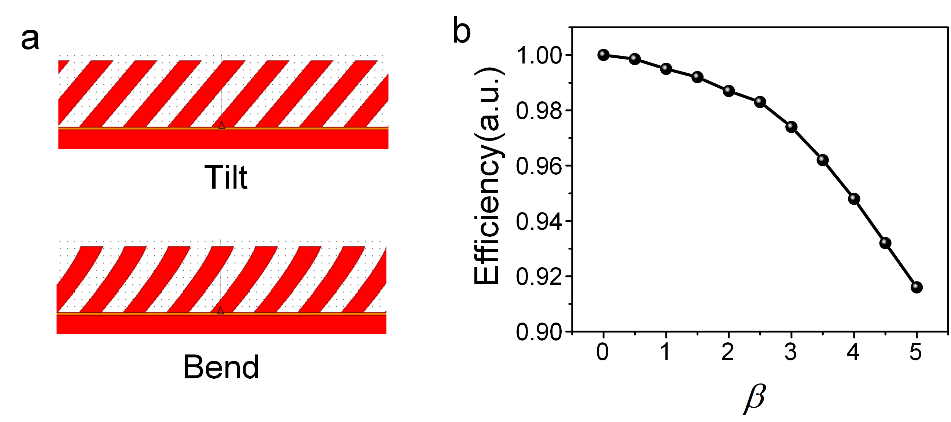
**

**Fig. S11** Simulation models of tilted nanograting structures and the influence of bending deformation on diffraction efficiency.

**Figure S11a** shows the model for the customized design of high-efficiency tilted gratings in Rsoft software, as well as the tilted gratings model with introduced bending deformation. The structures of these models have been divided into multiple layers to improve the simulation accuracy. **Figure S11b** shows the influence of bending deformation on the diffraction efficiency of the custom-designed high-efficiency tilted grating structures. It is evident that the greater the bending deformation, the lower the relative diffraction efficiency. Therefore, when fabricating high-angle-tilted structures, it is crucial to control the bending deformation within a reasonable range.

**S16 Geometric parameters and optical layout of the fabricated AR display**


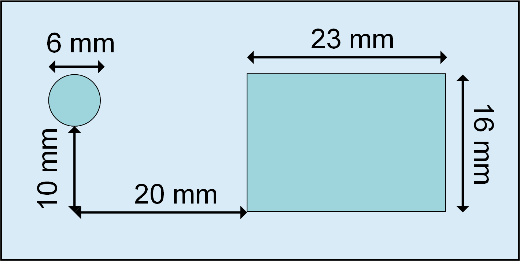


**Fig. S12** Top view and dimensions of the AR displays. The in-coupling region is a circular area with a diameter of 6 mm, integrating with the designed and fabricated tilted nanostructures that serve to couple optical signals from the engine into the waveguide. The in-coupling region is a rectangular output area measuring 23 mm × 16 mm, primarily enabling directional out-coupling of optical signals for display imaging.

**S17 Comparison of different AR display schemes**

We present a quantitative comparative analysis between the AR display developed in this work and other advanced grating structures previously reported for similar AR applications. This comparison encompasses key parameters including structural configuration, diffraction efficiency, material systems, and fabrication processes, as detailed in the **Table S2**.

**Table S2** comparison of different AR display schemes

| **References** | **Structure parameters** | | **Diffraction**  **efficiency** | **Materials** | **Fabrication methods** |
| --- | --- | --- | --- | --- | --- |
|  | **Structure morphology** | **Angle** |  |  |  |
| This paper |  | 40° | 85.6% | Polymer-TiO_2_ | E-G-N |
| [S19] |  | 35° | 80% | Polymer | Traditional nanoimprint |
| [S20] |  | 18° | 85% | MgF_2_+  SiO_2_ | EBL+etching |
| [S21] |  | 28.2° | 80.42% | SiO_2_ | EBL+etching |
| [S22] |  | 25° | ~ | Polymer | Traditional nanoimprint |
| [S23] |  | 20° | 85% | SiO_2_ | Simulation |
| [S24] |  | 25° | 43% | Polymer | Traditional  Nanoimprint |
| [S25] |  | 6.9° | 78.6% | SiO_2_ | EBL+etching |
| [S26] |  | 0° | 5% | Si_3_N_4_ | EBL+etching |
| [S27] |  | 0° | 25% | Si_3_N_4_ | EBL+etching |
| [S28] |  | 0° | 25% | SiC | EBL+etching |
| [S29] |  | 0° | 9% | Poly-Si | Transfer printing+etching |
| [S30] |  | 0° | 47.5 | SiO_2_ | EBL+etching |

Comprehensive comparative analysis reveals that tilted nanostructures exhibit significant advantages in diffraction efficiency, with the developed tilted gratings in this work demonstrating superior performance relative to peer studies. While other complex functional structures possess advantages for specialized optical applications such as achromatic applications, they rely on electron-beam lithography (EBL) and etching process. Limited by manufacturing costs and processing efficiency, these techniques can only achieve small-area sample preparation. Conversely, the proposed technique achieves a synergistic breakthrough across efficiency, cost, tilted-angle range, and processing area. Its wafer-scale fabrication capability provides a solution that balances large-area processing capacity and cost-effectiveness for the industrial-scale manufacturing of AR displays.

**S18 Reliability analysis of batch fabrication**


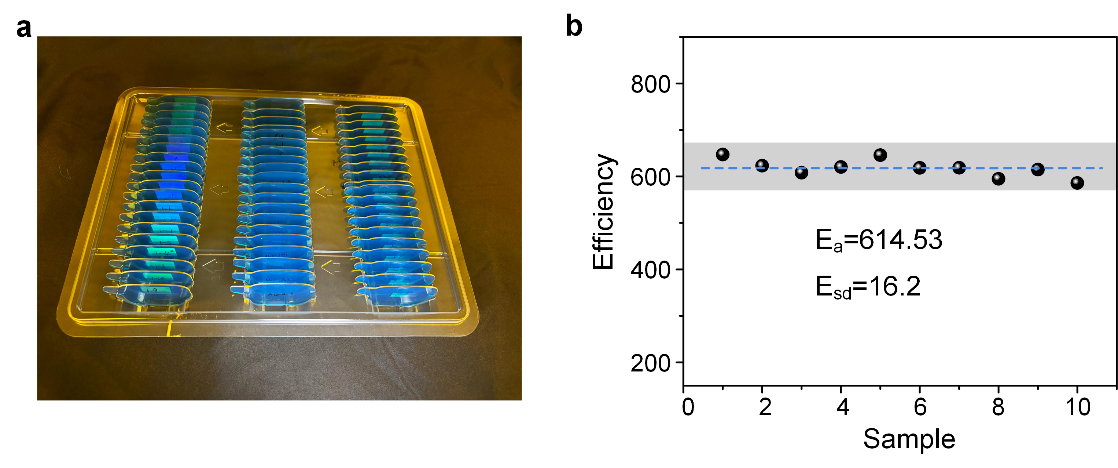


**Fig. S13** Reliability analysis of batch fabrication. **a** Batch-fabricated AR displays, **b** 10 samples were selected for optical performance characterization, and the results show a standard deviation of 16.2 for optical efficiency parameters, indicating minimal batch-to-batch variation and meeting the consistency requirements for scaled manufacturing.

**Supplementary References**

1. U. Tahir, M. Ahmad Kamran, M.Y. Jeong, Numerical study on the optimization of roll-to-roll ultraviolet imprint lithography. Coatings **9**(9), 573 (2019). <https://doi.org/10.3390/coatings9090573>
2. H. Wu, P. Yi, L. Peng, X. Lai, Study on bubble defects in roll-to-roll UV imprinting process for micropyramid arrays. I. Experiments. J. Vac. Sci. Technol. B Microelectron. Nanometer Struct. Process. Meas. Phenom. **34**(2), 021201 (2016). <https://doi.org/10.1116/1.4941445>
3. J.-K. Lee, S.-H. Lee, J.-H. Min, I.-Y. Jang, C.-K. Kim et al., Oblique-directional plasma etching of Si using a faraday cage. J. Electrochem. Soc. **156**(7), D222 (2009). <https://doi.org/10.1149/1.3122623>
4. D. Bruce Burckel, P.S. Finnegan, M. David Henry, P.J. Resnick, R.L. Jarecki, Oblique patterned etching of vertical silicon sidewalls. Appl. Phys. Lett. **108**(14), 142103 (2016). <https://doi.org/10.1063/1.4945681>
5. S.A. Cybart, P. Roediger, E. Ulin-Avila, S.M. Wu, T.J. Wong et al., Nanometer scale high-aspect-ratio trench etching at controllable angles using ballistic reactive ion etching. J. Vac. Sci. Technol. B Microelectron. Nanometer Struct. **31**(1), 010604 (2013). <https://doi.org/10.1116/1.4773919>
6. B. Chang, Oblique angled plasma etching for 3D silicon structures with wiggling geometries. Nanotechnology **31**(8), 085301 (2020). <https://doi.org/10.1088/1361-6528/ab53fb>
7. K. Kim, K. Park, H. Nam, G.H. Kim, S.K. Hong et al., Fabrication of oblique submicron-scale structures using synchrotron hard X-ray lithography. Polymers **13**(7), 1045 (2021). <https://doi.org/10.3390/polym13071045>
8. J. Zhang, B. Shokouhi, B. Cui, Tilted nanostructure fabrication by electron beam lithography. J. Vac. Sci. Technol. B Microelectron. Nanometer Struct. Process. Meas. Phenom. **30**(6), 06F302 (2012). <https://doi.org/10.1116/1.4754809>
9. S. Takahashi, K. Suzuki, M. Okano, M. Imada, T. Nakamori et al., Direct creation of three-dimensional photonic crystals by a top-down approach. Nat. Mater. **8**(9), 721–725 (2009). <https://doi.org/10.1038/nmat2507>
10. T. Levola, P. Laakkonen, Replicated slanted gratings with a high refractive index material for in and outcoupling of light. Opt. Express **15**(5), 2067–2074 (2007). <https://doi.org/10.1364/oe.15.002067>
11. T. Duan, X. Li, R. Wang, F. Chen, X. Qiao, Femtosecond laser plane-by-plane inscription of chirped and tilted fiber Bragg gratings. J. Light. Technol. **42**(17), 6083–6089 (2024). <https://doi.org/10.1109/JLT.2024.3403657>
12. J. Fantova, A. Rodríguez, L. Omeñaca, O. Beldarrain, G.G. Mandayo et al., Single-step fabrication of highly tunable blazed gratings using triangular-shaped femtosecond laser pulses. Micromachines **15**(6), 711 (2024). <https://doi.org/10.3390/mi15060711>
13. A. Toros, N. Restori, M. Kiss, T. Scharf, N. Quack, Single crystal diamond blazed diffraction gratings and Fresnel microlens arrays with improved optical performance by high-resolution 3D laser lithography and pattern transfer by dry etching. OSA Continuum **2**(12), 3374 (2019). <https://doi.org/10.1364/osac.2.003374>
14. S. Alamri, M. El-Khoury, A.I. Aguilar-Morales, S. Storm, T. Kunze et al., Fabrication of inclined non-symmetrical periodic micro-structures using Direct Laser Interference Patterning. Sci. Rep. **9**(1), 5455 (2019). <https://doi.org/10.1038/s41598-019-41902-x>
15. K. Sivashanmugan, J.-D. Liao, B.H. Liu, Focused-ion-beam-fabricated homogeneous acute-angled Au nanorods for surface-enhanced Raman scattering. Appl. Phys. Express **8**(5), 052402 (2015). <https://doi.org/10.7567/apex.8.052402>
16. Y. Chen, J. Gao, X. Yang, Chiral metamaterials of plasmonic slanted nanoapertures with symmetry breaking. Nano Lett. **18**(1), 520–527 (2018). <https://doi.org/10.1021/acs.nanolett.7b04515>
17. S.Yamashita, T.Oishi, T.Miyaji, C.Watanabe, N.Nitta, Tilted InSb nanostructures fabricated by off-normal ion beam irradiation. philos Mag Lett **98**(10), 446–455 (2018). <https://doi.org/10.1080/09500839.2019.1567947>
18. J. Lv, E.H. Khoo, E.S.P. Leong, L. Hu, X. Jiang et al., Maskless fabrication of slanted annular aperture arrays. Nanotechnology **28**(22), 225302 (2017). <https://doi.org/10.1088/1361-6528/aa6c95>
19. T. Levola, P. Laakkonen, Replicated slanted gratings with a high refractive index material for in and outcoupling of light. Opt. Express **15**(5), 2067–2074 (2007). <https://doi.org/10.1364/oe.15.002067>
20. W.-C. Liu, G. Jin, Y.-F. Xie, P. Sun, B. Zhou et al., Broadband high-efficiency polarization-independent double-layer slanted grating for RGB colors. Opt. Commun. **488**, 126864 (2021). <https://doi.org/10.1016/j.optcom.2021.126864>
21. J. Zhang, S. Liu, W. Zhang, S. Jiang, D. Ma et al., Design of waveguide with double layer diffractive optical elements for augmented reality displays. Sci. Rep. **14**(1), 24310 (2024). <https://doi.org/10.1038/s41598-024-75766-7>
22. X. Guo, Q. Song, S. Ma, J. Wang, G. Ma et al., Design and fabrication of polygonal grating waveguide display with full-color 2D eye-box expansion. Opt. Lasers Eng. **180**, 108311 (2024). <https://doi.org/10.1016/j.optlaseng.2024.108311>
23. C.P. Chen, X. Ma, S.P. Zou, T. Liu, Q. Chu et al., Quad-channel waveguide-based near-eye display for metaverse. Displays **81**, 102582 (2024). <https://doi.org/10.1016/j.displa.2023.102582>
24. B. Bai, J. Laukkanen, M. Kuittinen, S. Siitonen, Optimization of nonbinary slanted surface-relief gratings as high-efficiency broadband couplers for light guides. Appl. Opt. **49**(28), 5454–5464 (2010). <https://doi.org/10.1364/AO.49.005454>
25. C.-Y. Chen, Q.-L. Deng, B.-S. Lin, W.-C. Hung, Quartz-blazed grating applied on autostereoscopic display. J. Disp. Technol. **8**(8), 433–438 (2012). <https://doi.org/10.1109/JDT.2012.2194984>
26. Z. Tian, X. Zhu, P.A. Surman, Z. Chen, X.W. Sun, An achromatic metasurface waveguide for augmented reality displays. Light Sci. Appl. **14**(1), 94 (2025). <https://doi.org/10.1038/s41377-025-01761-w>
27. S. Moon, S. Kim, J. Kim, C.-K. Lee, J. Rho, Single-layer waveguide displays using achromatic metagratings for full-colour augmented reality. Nat. Nanotechnol. **20**(6), 747–754 (2025). <https://doi.org/10.1038/s41565-025-01887-3>
28. Y. Li, S. Chen, H. Liang, X. Ren, L. Luo et al., Ultracompact multifunctional metalens visor for augmented reality displays. PhotoniX **3**(1), 29 (2022). <https://doi.org/10.1186/s43074-022-00075-z>
29. G.-Y. Lee, J.-Y. Hong, S. Hwang, S. Moon, H. Kang et al., Metasurface eyepiece for augmented reality. Nat. Commun. **9**(1), 4562 (2018). <https://doi.org/10.1038/s41467-018-07011-5>
30. J. Xiao, J. Liu, J. Han, Y. Wang, Design of achromatic surface microstructure for near-eye display with diffractive waveguide. Opt. Commun. **452**, 411–416 (2019). <https://doi.org/10.1016/j.optcom.2019.04.004>
